# Identification of Genomic Regions Associated with Agronomic and Disease Resistance Traits in a Large Set of Multiple DH Populations

**DOI:** 10.3390/genes13020351

**Published:** 2022-02-15

**Authors:** Kassahun Sadessa, Yoseph Beyene, Beatrice E. Ifie, L. M. Suresh, Michael S. Olsen, Veronica Ogugo, Dagne Wegary, Pangirayi Tongoona, Eric Danquah, Samuel Kwame Offei, Boddupalli M. Prasanna, Manje Gowda

**Affiliations:** 1 Ethiopian Institute of Agricultural Research (EIAR), Ambo Agricultural Research Center, Ambo P.O. Box 37, West Shoa, Ethiopia; kbsadessa@wacci.ug.edu.gh; 2 International Maize and Wheat Improvement Center (CIMMYT), ICRAF House, P.O. Box 1041-00621, Nairobi 00100, Kenya; y.beyene@cgiar.org (Y.B.); l.m.suresh@cgiar.org (L.M.S.); m.olsen@cgiar.org (M.S.O.); v.ogugo@cgiar.org (V.O.); b.m.prasanna@cgiar.org (B.M.P.); 3 International Maize and Wheat Improvement Center (CIMMYT), 12.5 KM Peg, Harare P.O. Box MP163, Zimbabwe; d.wegary@cgiar.org; 4 West Africa Centre for Crop Improvement (WACCI), College of Basic and Applied Sciences, University of Ghana, Legon, P.O. Box LG23, Accra 00233, Ghana; bifie@wacci.ug.edu.gh (B.E.I.); ptongoona@wacci.ug.edu.gh (P.T.); edanquah@wacci.ug.edu.gh (E.D.); offei@wacci.ug.edu.gh (S.K.O.)

**Keywords:** genome-wide association study, genomic prediction, water stress, well-watered, maize lethal necrosis, genotyping by sequencing, well-watered, water stress

## Abstract

Breeding maize lines with the improved level of desired agronomic traits under optimum and drought conditions as well as increased levels of resistance to several diseases such as maize lethal necrosis (MLN) is one of the most sustainable approaches for the sub-Saharan African region. In this study, 879 doubled haploid (DH) lines derived from 26 biparental populations were evaluated under artificial inoculation of MLN, as well as under well-watered (WW) and water-stressed (WS) conditions for grain yield and other agronomic traits. All DH lines were used for analyses of genotypic variability, association studies, and genomic predictions for the grain yield and other yield-related traits. Genome-wide association study (GWAS) using a mixed linear FarmCPU model identified SNPs associated with the studied traits i.e., about seven and eight SNPs for the grain yield; 16 and 12 for anthesis date; seven and eight for anthesis silking interval; 14 and 5 for both ear and plant height; and 15 and 5 for moisture under both WW and WS environments, respectively. Similarly, about 13 and 11 SNPs associated with gray leaf spot and turcicum leaf blight were identified. Eleven SNPs associated with senescence under WS management that had depicted drought-stress-tolerant QTLs were identified. Under MLN artificial inoculation, a total of 12 and 10 SNPs associated with MLN disease severity and AUDPC traits, respectively, were identified. Genomic prediction under WW, WS, and MLN disease artificial inoculation revealed moderate-to-high prediction accuracy. The findings of this study provide useful information on understanding the genetic basis for the MLN resistance, grain yield, and other agronomic traits under MLN artificial inoculation, WW, and WS conditions. Therefore, the obtained information can be used for further validation and developing functional molecular markers for marker-assisted selection and for implementing genomic prediction to develop superior elite lines.

## 1. Introduction

Maize is an important staple food crop in sub-Saharan Africa (SSA) where a large area is under maize production [1]. In east Africa, 82.48 million hectares (m ha) were covered by maize and about 156.21 million tons of maize grain were produced with productivity of 1.89 tons per ha (http://www.fao.org/faostat/, accessed on 2 November 2021). Both biotic and abiotic stresses are the major threats to crop production, particularly maize in SSA. Drought stress, high costs of improved seeds and fertilizers [2], and biotic stresses such as maize lethal necrosis (MLN) disease are the limiting factors for maize production in east Africa.

MLN was first reported in Kenya in 2011 and later reported in Tanzania, Uganda, Rwanda, D.R. Congo, and Ethiopia [3,4,5]. *Maize chlorotic mottle virus* (MCMV) and *sugarcane mosaic virus* (SCMV) viruses were the confirmed pathogens that have jointly incited the MLN disease [5,6,7]. Both MCMV and SCMV are transmitted by insect vectors (MCMV by thrips and semipersistent beetles; SCMV by aphids) [5,8]. MCMV has been confirmed for its transmission by seeds and infected soils, making the management of MLN more challenging [6,9,10,11]. Based on the maize plant growth stages and environment conduciveness for MLN causing pathogens, the yield losses ranged from 30–100% [12]. Thus, the management of MLN demands proper identification of resistant germplasm sources and associated genes or quantitative trait loci (QTL) that aid to develop the resistant hybrids or varieties [13].

Doubled haploid (DH) lines allow complete homozygosity over lines developed through pedigree breeding; this allows precision in phenotyping over multiple locations and years [14]. Further, high genetic variance in DH lines enhances response to selection [15] by increasing heritability for various traits. Compared to breeding under well-watered (WW) conditions, the genetic variability, trait heritability, disease resistance, and selection gain are very low for breeding under water stress (WS) conditions [16]; thus, WS condition makes the identification of best genotypes and expression of complex traits. These challenges are designed to be solved through established managed drought tolerance and disease screening facilities, not to lose the genetic variations, and to produce good yield under stress conditions. Understanding the maize crop’s behavior under WS for grain yield and yield-related traits, proper statistical design and breeding scheme help to select the best genotypes under WS environments [16,17].

Advancement in next-generation sequencing tools promoted genome-wide association studies (GWAS) in many crops including maize [18]. Association analysis is based on the non-random association between genotypes and phenotypes of the diverse distantly related individuals [19]. The significance of the marker–phenotype association could be declared when the marker polymorphism is located within the linkage disequilibrium (LD) region. To detect an association of complex traits, a minimum LD average with cut-off point of r^2^ =0.1 was used [19]. In maize, the rate of LD decay approximated to 1, 2, and 200–500 kb in landraces, diverse inbred lines, and commercial elite inbred lines, respectively [20].

GWAS is useful in allele mining by dissecting the quantitative traits [19]. QTL or gene mapping consists of linkage map construction and identifying genomic regions associated with the targeted QTL [21]. QTL mapping helps to understand the genetic inheritance of quantitative traits [22,23]. Breeding for drought tolerance is complex since the trait is influenced by the environment and many genes with small effects [24]. In maize, about 239 QTLs related to drought tolerance were reported [25,26]. Five drought-tolerant QTLs closely linked to grain yield were reported by Agrama and Moussa [27]. Semagn et al. [2] reported four meta-QTLs associated with grain yield for both under drought and optimum management. The high QTL detection power and fine resolution of mapping are exploited by joint linkage association mapping in multiple biparental populations [28,29,30]. The identification and validation of novel genomic regions associated with economically important traits under WW and WS as well as MLN are important to accelerate the development of climate-resilient improved maize varieties to enhance high maize productions in smallholder families and contribute to food security [12,31,32].

Genomic selection (GS) uses genome-wide markers to predict the breeding values of individuals by trapping the effects of both major and minor genes [33]. In GS, from the training population, the effect of all markers are estimated, and then the genomic estimated breeding values (GEBVs) of the untested but genotyped lines are computed [33]. Lines in the testing population are only genotyped, not phenotyped, and thus important in reducing the breeding cycle and increasing the genetic gain per unit time. GS is effective in several crops over a wide range of marker densities, trait complexities, and breeding populations [34,35,36], where varying levels of prediction accuracy have been achieved in different studies.

To understand how WS affects grain yield and other key traits, this study was performed using a tropical maize population under drought and optimum conditions across multi-location field trials and the MLN effect under artificial inoculation in Kenya. The objectives of the study were to (i) evaluate the large set of 879 tropical and subtropical maize DH lines for their responses to MLN disease severity under artificial inoculation, grain yield (GY), and other yield-related traits under WW and WS conditions; (ii) identify genomic regions and putative candidate genes associated with these traits across the three management conditions; and (iii) assess the potential of GS within management conditions. This study will provide valuable information for uncovering the genetic basis of GY under WW and WS conditions.

## 2. Materials and Methods

### 2.1. Plant Materials and Field Trials

In this study, 1462 DH lines from 40 populations were phenotyped in multiple locations under WW, WS, and MLN artificial inoculation conditions. Whereas, among these DH lines, 879 DH lines derived from 26 DH populations were genotyped, for the final analyses, we used only 879 DH lines. There are 26 parental lines were used to develop these DH populations (Supplementary Table S1). Among these, three lines with LapostaSequiaC17 background are known for their drought tolerance, whereas other CIMMYT maize lines such as CML312, CML395, CML442, CML444, and CML539 are commonly used as parents for most of single cross testers of most of commercial hybrids released in east and southern Africa. Additionally, new lines, which showed a better level of resistance for foliar diseases, were also used in a way to bring both biotic and abiotic stress tolerant lines together in a set of lines. The DH lines were crossed to a single cross tester from the opposite heterotic group. All DH lines were formed 17 sets and planted as 17 trials. There were seven commercial checks used, which were repeated in each trial, acting as connecting genotypes for each trial. Both genotypes and checks were replicated two times. The trials were connected by common checks (DK8031, H517, Pioneer30G19, PAN4M19, DUMA43, DH04, and WE1101). All the DH lines were evaluated in 17 connected trials under WW (Kakamega and Kiboko), WS (Kiboko), and MLN (Naivasha) conditions in Kenya. A single row of the plot with 4 m length in two replications was arranged in an α lattice design. Two seeds were planted per hill and thinned to one while 75 cm spacing between rows and 25 cm between plants was used. Eleven commercial checks were used in each trial. All recommended agronomic practices were applied uniformly to each trial.

### 2.2. Mass Production and Artificial Inoculation of MLN Viruses

The detailed protocol on the preparation of inoculum is explained in earlier studies [12,13,37]. In brief, the stock isolates of MCMV and SCMV pathogens were mass-produced in separately managed greenhouses. The sap extraction of both MCMV and SCMV pathogen inoculum was made using 0.1 mM potassium–phosphate and pH 7.0 extraction buffers in 1:10 ratio and mixed at a ratio of 4 SCMV:1 MCMV to create MLN disease inoculum. The inoculum was sieved using cheesecloth, and then carborundum was added to the mixture of MLN inciting pathogen inoculum at the rate of 0.02 g/mL to create a wound that enhances an attachment and penetration of the virus particles into the host plant. Before field inoculation, the mixture of MCMV and SCMV virus inoculum was checked using the target pathogen-specific antibodies using enzyme-linked immuno-sorbent assay (ELISA). Field inoculation was performed using a backpack motorized knapsack sprayer at the four weeks of plant growth stage after planting, and the second inoculation was made one week after the first spray to keep a uniform inoculation. Two weeks after the second inoculation, the establishment, development, and existence of the MLN disease-causing viruses were rechecked using ELISA kits. The MLN disease severity (MLN-DS) rating scale of 1–9 was used, where 1 is highly resistant with no MLN disease symptoms and 9 is highly susceptible or necrosis symptoms or total death of the plant. The MLN disease data were recorded four times at ten-day- intervals starting from the third week of the post-inoculation. The progress of MLN disease or area under the disease progress curve (AUDPC) was calculated from the recorded MLN-DS data over four time intervals [5,32,38].

### 2.3. Phenotyping and Data Analysis

The DH lines were evaluated at Kakamega under WW management, Kiboko under both WW and WS at different sites, and Naivasha under artificial inoculation of MLN for two seasons. Grain yield (GY, ton/ha), anthesis date (AD, 50% of pollen shed), silking date (SD, 50 % of silking), anthesis silking interval (ASI, the difference between anthesis and sinking dates), plant height (PH, cm) measured from the ground level to the base of the tassel after milk stage, ear height (EH, cm) measured from the ground level to the node bearing the uppermost ear after milking stage, moisture (MOI, percent moisture content of the grain at the time of harvesting using moisture meter), senescence (SEN, percent leaves lost chlorophyll to green leaves at the mid-silking), grey leaf spot (GLS, recorded using 1–9 scale), turcicum leaf blight (TLB, measured using 1–9 rating scale), common rust (CR, recorded using 1–9 rating scale), and MLN-DS (measured using the rating scale of 1 to 9 score) were recorded and analyzed. All the traits were phenotyped in all the trials but not in all the management conditions. For example, AD, ASI, EH, PH, MOI, and GY traits were phenotyped under both WW and WS; TLB, GLS, and CR under WW; SEN and ER under WS; and MLN-DS and AUDPC under MLN management conditions.

Statistical model fitting for different traits was checked by plotting the histogram with standardized residuals. A plot of residuals against fitted values has shown that the residuals were symmetrically distributed with constant variance for all traits; thus, the data were not transformed. The phenotypic traits were analyzed with the restricted maximum likelihood (REML) method designed in the multi-environment trial analysis (META) R software developed in CIMMYT [39]. The following mixed model was used for across environments data analyses.

$$Y_{ijkl}=\mu +G_{i}+L_{j}+{\left(GL\right)}_{ij}+R{\left(L\right)}_{kj}+B{\left(RL\right)}_{ljk}+e_{ijkl}\;$$

where: $Y_{ijkl}$ is the phenotypic observation at the *i*th genotype, *j*th environment in *k*th replication of the *l*th incomplete block, *μ* is overall means, *G_i_* is the genetic effect of the *i*th genotype, *L_j_* is the effect of the *j*th environment, ${\left(GL\right)}_{ij}$ is genotype by environment interaction, $R{\left(L\right)}_{kj}$ is the effect of the *k*th replication at the *j*th environment, $B{\left(RL\right)}_{ljk}$ is the effect of the *l*th incomplete block in the *k*th replication at the *j*th environment, and $e_{ijkl}$ is the residual. The selected traits’ broad-sense heritability (H^2^) was calculated as follows:

$$H^{2}=\sigma_{G}^{2}/(\sigma_{G}^{2}/\left(\sigma_{G}^{2}+\frac{\sigma_{GxE}^{2}}{L}+\frac{\sigma_{e}^{2}}{LR}\right)),$$

where $\sigma_{G}^{2}$, $\sigma_{GxE}^{2}$, $\sigma_{e}^{2}$, L, and R referred to the genotypic, genotype by environment interaction, error variance, environment, and replication, respectively. Best linear unbiased estimates (BLUEs) and best linear unbiased predictions (BLUPs) for all traits were calculated. The traits phenotypic distribution and Pearson’s correlation coefficient were performed and displayed using R scrips (http://www.R-project.org, accessed on 17 November 2021).

### 2.4. Genotypic Data Analyses

All 879 DH lines were genotyped with a high-density genotype by sequencing (GBS) platform using the pre-developed protocol at the Institute for Genomic Diversity, Cornell University, Ithaca, USA [2,31,40]. DNA was extracted from the young leaves using the cetyltrimethylammonium bromide (CTAB) method [41]. Raw GBS data had a total of 955,120 SNPs loci distributed across maize genome. The raw GBS SNPs data were imputed by default parameter filling methods [24,42]. Different filtering criteria were applied to the raw data to obtain input data for LD and GWAS analyses. For LD, the raw data were filtered based on no missing data and >10% minor allele frequency (MAF). The BLUPs for the selected traits (MLN-DS, AUDPC, AD, ASI, GY, EH, MOI, SEN, and PH) across environments were used for the GWAS study. SNPs quality screening was performed using trait analysis by association, evolution, and linkage (TASSEL v.5.2.24) software [43] by filtering and discarding the SNPs with a <0.05 of MAF and heterozygosity of >0.05, resulted into 226,940 SNPs. SNPs and physical distance between SNPs were used to detect genome-wide LD [44]. LD decay was calculated at r^2^ = 0.2 and r^2^ = 0.1 using average pairwise distance, where the nonlinear model r^2^ was used [45,46]. Scatter plots and fitted smooth curves for estimating LD decay were plotted using the LOESS function in R [47].

### 2.5. Population Structure and GWAS

The genetic relationship tree construction for 879 DH lines was performed using Darwin 6.0.21 software. At the first step, the genetic distance matrix was calculated based on the mean Euclidean method where homozygote 100 and heterozygote 50% similarity were considered. Secondly, the unweighted neighbor-joining clustering method was employed to construct the diversity tree of the genotypes. The population structure of 879 DH lines, which had both phenotypic and genotypic data, were analyzed and sub-grouped using structure software 2.3.4 version with 6745 SNPs [48,49] based on the variability of allele frequencies both within and between populations genetic distance. The number of discontinuous population structure clusters (K) was predicted from one to five with ten iterations. The true number of population structure clusters (delta K value) were harvested online using an available structure harvester software based on the highest ln P(D). The unique population genetic subcluster was represented by each color bar at a *p* = 0.001. The period of length of burn-in was set to 10,000, and Markov Chain Monte Carlo (MCMC) values were set to 10,000 cycles [48].

GWAS analysis was performed with the R package “FarmCPU—Fixed and random model Circulating Probability Unification” [50]. GBS marker data in the “hapmap” format were converted to numeric (0, 1, 2) with the “GAPIT” package [51]. The first three principal components (PCs) obtained from TASSEL [43] were used as an input for GWAS in FarmCPU. The kinship matrix was calculated with the default kinship algorithm. The analysis was performed with a maxLoop of five, *p* threshold of 0.01, a quantitative trait nucleotide (QTN) threshold of 0.01, and a MAF threshold of 0.05. The maxLoop refers to the total number of iterations used. The *p* values selected into the model for the first iteration, the *p*-value selected into the model from the second iteration, and the minimum MAF of SNPs used in the analysis refers to the *p* threshold, QTN threshold, and MAF threshold, respectively. To determine the significance threshold, multiple testing correction was conducted where the total number of tests was estimated based on the average extent of LD at r^2^ = 0.1. Concerning the above, the significant associations were declared when *p* values in independent tests were less than 9 × 10^−6^ [38,52]. The Blast search against maize reference genome “B73” was performed for the significant SNPs; subsequently, the candidate gene adjacent or exactly in the same position with the significant SNPs identified and annotated and the candidate gene biological function described for each of the studied target traits (http://blast.maizegdb.org/home.php, accessed on 23 November 2021; http://www.maizegdb.org, accessed on 23 November 2021). CurlyWhurly Version 1.19 was used to plot and visualize the first three analyzed PCA components (https://ics.hutton.ac.uk/curlywhirly/, accessed on 19 November 2021).

### 2.6. Genomic Predictions

The phenotypic traits BLUEs were used for the GS analysis. Ridge-regression BLUP (RR-BLUP) with five-fold cross-validation was applied. From the GBS data, a subset of 6745 SNPs distributed uniformly across the genome, with no missing values, and minor allele frequency >0.10 were used for GS in GWAS panel under different management conditions. Details of the implementation of the RR-BLUP model are described in Zhao et al. [36]. We applied a five-fold cross-validations ‘within population’ approach, where both training and estimation sets were derived from within the association panel under different management conditions. The prediction accuracy was calculated as the correlation between genomic estimated breeding values (GEBVs) and the observed phenotypes. A sampling of the training and validation sets was repeated 100 times for each approach.

## 3. Results

### 3.1. Phenotypic Variations and Correlations

**Figure 1:**
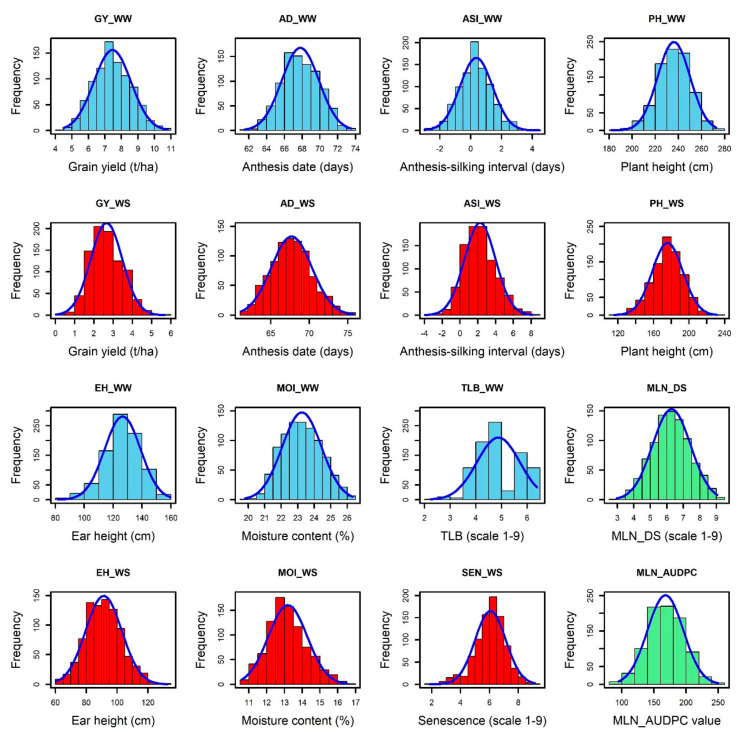
Phenotypic distributions of MLN_DS, MLN_AUDPC, AD, ASI, EH, PH, and MOI and TLB, SEN, and GY traits were measured under WW (blue), WS (red), and MLN (green) management. AD—anthesis date; ASI—anthesis silking interval; AUDPC—area under disease progress curve; EH—ear height; GY—grain yield; MLN_DS—maiz lethal necrosis disease severity; MOI—moisture content; PH—plant height; SEN—senescence; and TLB—turcicum leaf blight; WW—well watered; WS—water stressed.

**Figure 2:**
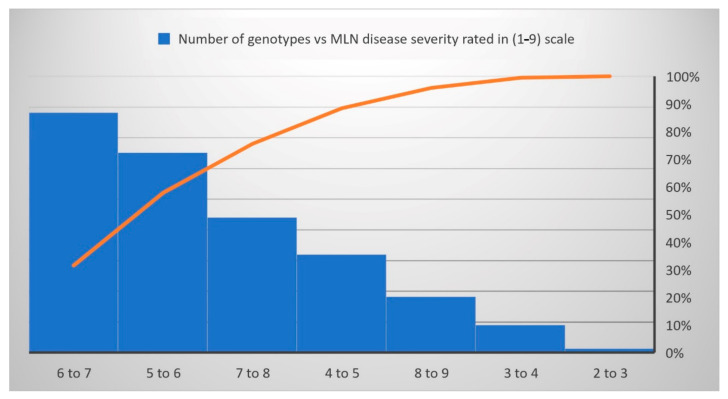
The reaction responses of 879 DH lines to MLN disease under artificial inoculation. MLN-DS rating scale (1–9) has been converted to a percentage.

The normal distribution was observed for each trait under WW and WS conditions (Figure 1). The analysis of variance revealed significant genotypic variance (Table 1) for the studied traits: MLN-DS and AUDPC under MLN management and GY, AD, ASI, PH, EH, TLB, MOI, CR, SEN, and GLS traits measured under WW and/or WS management. The variation for GY ranged from 4.35 to 11.66 tons/ha (mean = 7.54 t/ha) under WW condition and from 0.03 to 5.67 tons/ha (mean = 2.7 t/ha) under WS conditions (Table 1). The mean performance for AD showed 0.41 days earliness under WS compared to WW conditions. The range is higher for ASI under WS (−3.95 to 8.17 days) compared to WW (−3 to 4.5 days) conditions. The mean of PH and EH were reduced significantly under WS compared to WW conditions. Further, the range of distribution reduced drastically for SEN under WS. The META R combined analyses result revealed that the studied genotypes had a wide range of responses against the MLN-DS ranging from 2 to 9 (Figure 2, Table 1). GY had moderate broad-sense heritability (H^2^) under both WW and WS conditions, while MLN-DS and TLB had relatively high H^2^ with 0.67 and 0.80, respectively.

Under WS management, the genotypes CKDHL140940, CKDHL142056, CKDHL142091, CKDHL141377, and CKDHL142061 had produced the highest GY values of 5.67, 4.91, 4.85, 4.81, and 4.77 t/ha, respectively. The genotype CKDHL140037 had a lesser AD (62.11 days) than the grand mean (67.55 days) and the best check Duma43 (62.68 days). Genotype CKDHL140091 has shown 0.38 days less than the best check (0.74 days) and grand mean (2.28 days). Comparatively, plant height (185.55 cm), which is not too tall or short, was obtained in the genotype CKDHL140125, even though it was slightly higher than the best check KD8031 (183.88 cm) and grand mean (176.40 cm). Under WW management, the genotype CKDHL141097 was performed better than the best check CML444 and overall mean, which had the GY values of 9.17, 7.20, and 8.28 t/ha, respectively. An AD (65.93 days) was recorded in the CKDHL140933 genotype, which was earlier than the overall mean (67.78 days) and comparable to the best check Duma43 (64.47 days) under WW management. Similarly, the genotype CKDHL140876 had a good ASI of 0.37 days lesser than the grand mean (0.42 days) but not better than the check CZL04003 (0.35 days). About 52 DH lines were rated from two to four and have depicted the resistance reactions against the MLN-DS, while 735 DH lines rated from four to seven were grouped as moderately resistant, and the remaining 92 DH lines had seven to nine values and were grouped as susceptible genotypes (Figure 2).

**Figure 3:**
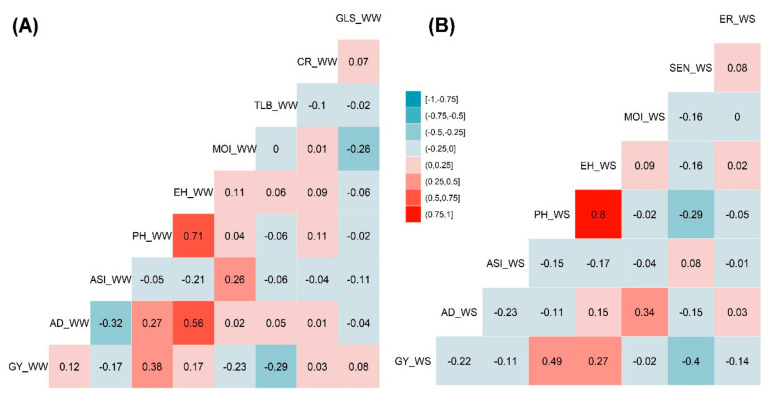
Pearson’s correlation of the phenotypic traits measured under WW (**A**) and WS (**B**) managements. The correlation level is color-coded according to the color key plotted in the center. Correlations with >0.10 and >0.15 were significant at 0.05 and 0.01 levels, respectively. AD-anthesis date; ASI-anthesis silking interval; CR-common rust; EH-ear height; ER-ear rot; GLS-gray leaf spot; GY-grain yield; MOI-grain moisture; PH-plant height; SEN-senescence; and TLB-turcicum leaf blight.

The phenotypic traits correlation analysis was performed independently for genotypes evaluated under both WW and WS management conditions. Significant strong positive correlations were observed between PH and EH both under WW and WS management, which was 0.71 and 0.80, respectively (Figure 3). EH and PH traits have revealed a positive correlation with GY that had the correlation values of 0.17 and 0.38 under WW management and 0.27 and 0.49 under WS management, respectively (Figure 3A,B). This positive correlation has indicated that an increase in EH and PH increased the GY to certain extent. AD had significant positive correlations with ER under WW (0.56) and MOI under WS (0.34) managements. Both AD and ASI were negatively correlated both under WW (−0.32) and (−0.23) WS environments. TLB under WW and SEN under WS management had slight negative correlations of −0.29 and −0.40, respectively, with GY that may have played a vital role in proportional yield reduction. Similarly, SEN had negative correlation (−0.29) with PH under the WS environment. MOI has depicted a negative correlation (−0.23) under WW with GY (Figure 3A,B).

### 3.2. Genetic Relationship, Population Structure, and Linkage Disequilibrium

The selected markers distribution was graphically presented in Supplementary Figure S1. A kinship matrix was developed that depicts the relatedness among the used DH lines (Supplementary Figure S2). Population relationship analyses in Darwin’s software had displayed the neighbor-joining of 879 DH lines dissimilarity tree, which was constructed based on the genetic distance matrix of 0.01 calculated by the Euclidean method. The total populations were clustered into three main diverse groups with many subtrees (Supplementary Figure S3). The first population diversity group had DH lines derived from CML395/CML505, which has contained about 440 individuals represented by red, the second group had LaPostaSeq C7-F64 as a common parent with 174 individuals represented by blue, and the third group had DH lines having one of either LaPostaSeq C7-F86 or LaPostaSeq C7-F18 as common parent with 265 DH lines represented by a purple color (Supplementary Figure S3).

**Figure 4:**
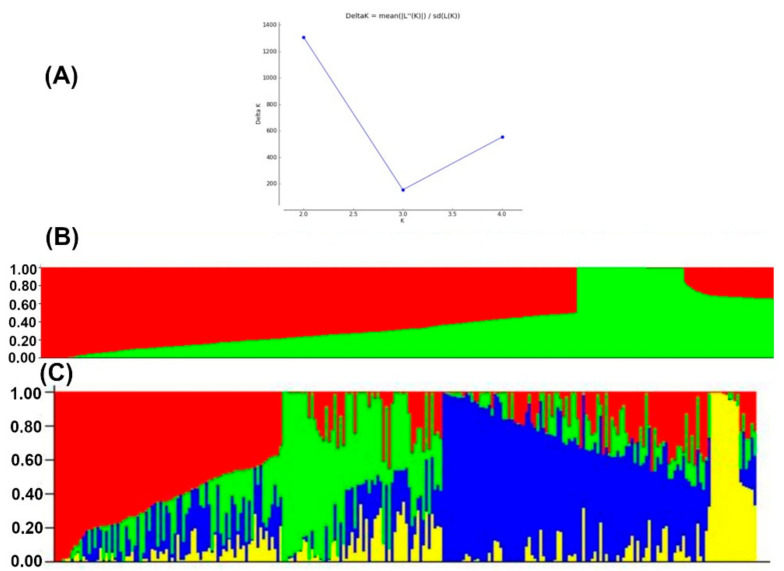
The four sub-populations of the 879 DH lines using GBS markers. (**A**) Best delta K estimation by Evanno method. (**B**) Estimated population structure of 879 tropical maize DH lines as revealed by GBS SNP markers for K = 2 and (**C**) for K=4. Blue, green red and yellow color represents sub-population 1, 2, 3, and 4, respectively.

**Figure 5:**
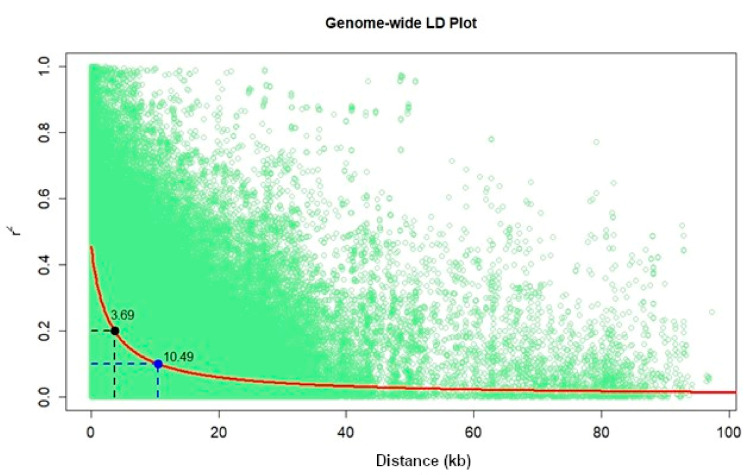
Genome-wide markers linkage disequilibrium (LD) plot representing the average pairwise distances genome-wide LD decay at r^2^ = 0.2 and r^2^ = 0.1 in set of DH populations with 879 lines.

Population structure analyses revealed delta K probability value with three to four clusters of 879 DH lines based on the highest ln P(D) values (Figure 4). An Evanno table was constructed in the structure harvester with the highest values of 204,444.45 ln P(K), 156.41 standard deviations ln P(K), and 1307.01 delta K. The delta K value-based line plot had suggested that the population could be structured into two to four groups (Figure 4). Pair-wise markers LD decay was measured as the r^2^ and plotted against their distance. LD sliding window type with 11,225 comparisons were obtained from adjacent markers, while each dot represented a pair of distances between two markers on the window and their squared correlation coefficient. The LD decay cut-off point (r^2^) at 0.2 and 0.1 had 3.69 and 10.49 Kbs average physical distance, respectively (Figure 5).

Based on principal component analyses all the DH lines were broadly categorized into three groups: Category One (215), Category Two (223), and Category Three (441) based on the displayed plot (Supplementary Figure S4). The populations CML444/LaPostaSeqC7-F64, CML395/CML505, CML538/LaPostaSeq C7-F18, CML442/ LaPostaSeqC7-F86, CML537/LaPostaSeqC7-F18, CML539/LaPostaSeqC7-F64, CML442/INTA-F2-192, and CML539/INTA-F2-192 were grouped into Category One, while CZL04003/LaPostaSeqC7-F18, CML538/ LaPostaSeqC7-F64, INTA-F2-192/LaPostaSeqC7-F18, CML537/LaPostaSeqC7-F86, CML536/LaPostaSeqC7-F18, CML539/LaPostaSeqC7-F64, CZL04003/LaPostaSeqC7-F86, CML445/LaPostaSeqC7-F64, CML536/LaPostaSeqC7-F64, CML444/LaPostaSeqC7-F86, CML312/LaPostaSeqC7-F64, and CML538/ CL-G1628 G16BNSeqC0F118 populations were grouped under Category Two; similarly, LPSC7-F180/ Katumani, Ry x CML395, WL429-40/[CML444/DRB-F2//DTPWC8F3], CML395/CML505, WL429-40/ [CML444/DRB-F2//DTPWC8F3], L118 6/[CML312/CML444//[DTP2WC4H255/LATA-F2], and I-38 x CML442 populations were grouped into Category Three (Supplementary Figure S4 and Supplementary Table S1). The first four principle components explained 10.51%, 7.63%, 6.16% and 3.19% of the total variation (Supplementary Figure S4).

### 3.3. GWAS Results

**Figure 6:**
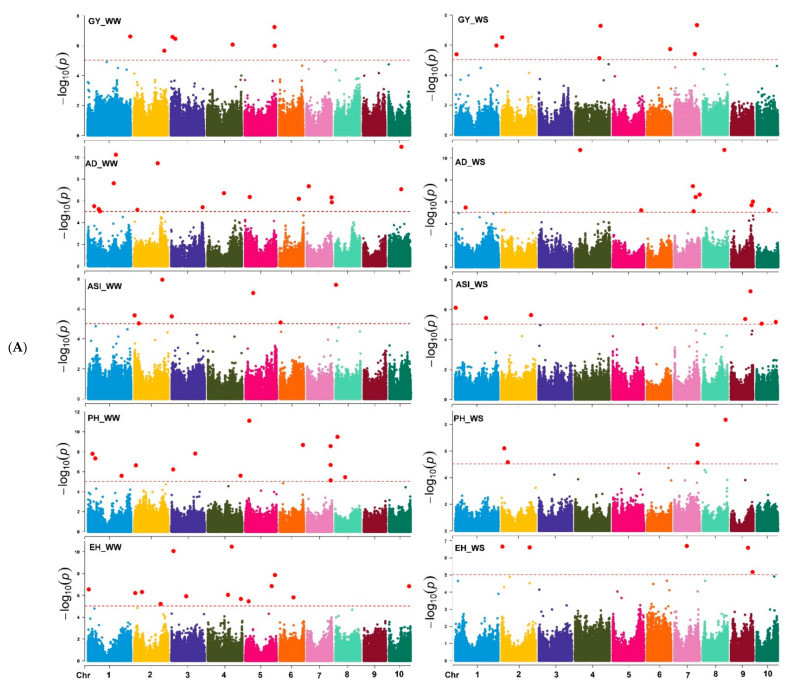

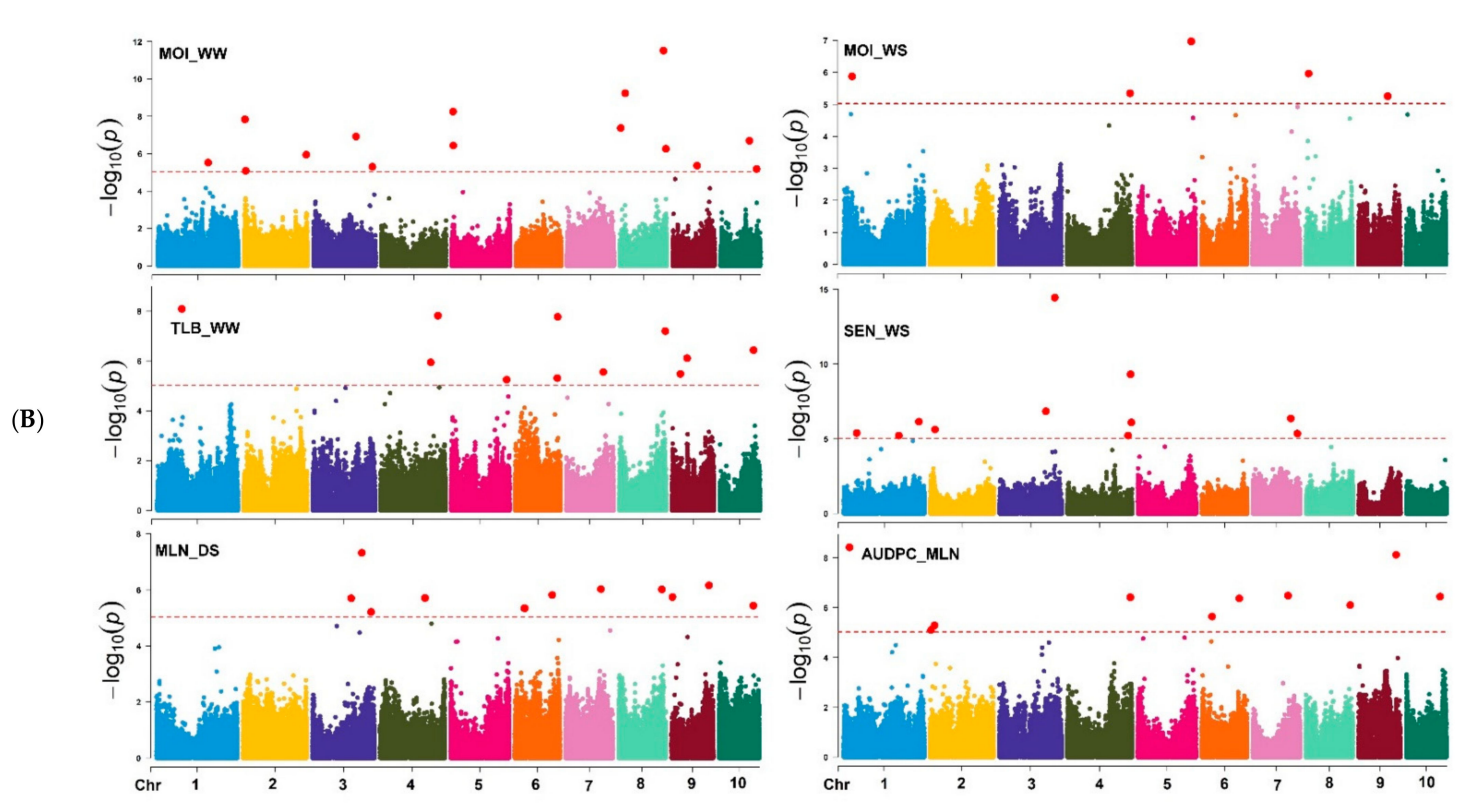
(**A**) Manhattan plots for GY and other related traits measured under WW, and WS managements. The X-axis shows the SNPs position on the chromosome, and the Y-axis shows the negative log base 10 of the *p*-values; for ease of discrimination, each chromosome was colored differently. The horizontal line portrays the significance threshold (marker *p*-value < 9 × 10^−6^). AD*-*anthesis date, ASI*—*anthesis silking interval, EH*—*ear height, GY*—*grain yield, PH*—*plant height, WW*—*well-watered, WS*—*water-stressed. (**B**) Manhattan plots for MOI, TLB, SEN, MLN-GS and AUDPC measured under WW, WS, and MLN managements. The X-axis shows the SNPs position on the chromosome, and the Y-axis shows the negative log base 10 of the *p*-values; for ease of discrimination, each chromosome was colored differently. The horizontal line portrays the significance threshold (marker *p*-value < 9 × 10^−6^). AUDPC*—*area under disease progress curve, MLN-DS*—*maize lethal necrosis disease severity, MOI*—*grain moisture, TLB*—*turcicum leaf blight, SEN—senescence, WW*—*well-watered, WS*—*water-stressed.

Based on the marker *p*-value at the significance threshold cut-off (*p* = 9 × 10^−6^), the marker positions, putative candidate gene, and its biological function were annotated for each trait. The GWAS results for all traits are summarized using Manhattan plots (Figure 6A,B) and QQ plots (Supplementary Figure S5). The GWAS analyses identified SNPs associated with the studied traits, i.e., 7 and 8 SNPs were associated with GY; 16 and 12 SNPs with AD; 7 and 8 SNPs with ASI; 14 and 5 SNPs with EH; 14 and 5 SNPs with PH; and 15 and 5 with MOI under WW and WS management, respectively (Table 2 and Supplementary Table S2). Similarly, 14 and 11 SNPs were associated with GLS and TLB resistance under WW environments, respectively. Under the WS environment, 11 SNPs were associated with SEN, whereas 12 and 10 SNPs associated with MLN-DS and AUDPC traits, respectively, under MLN artificial inoculation (Table 2 and Table 3). Some of the SNPs that had the highest significance value were closely associated with the putative genes governing the studied target traits (Supplementary Table S2).

The most closely associated and identified SNPs to the studied traits on different chromosomes and their position on the chromosomes are the SNPs *S5_206615806* and *S7_157468954* on chromosomes 5 and 7 linked to GY (Table 4), *S8_148392640* and *S10_88394535* on chromosomes 8 and 10 linked to AD; *S2_194040196* and *S9_136924349* on chromosomes 2 and 9 to ASI (Table 5), *S5_27226539* and *S8_158986117* on chromosomes 5 and 8 to PH, *S4_166924899* and *S7_87194068* on chromosomes 4 and 7 to EH (Table 6), and *S8_162561752* and *S5_200299111* on chromosomes 8 and 5 to MOI (Supplementary Table S2) under WW and WS environments. The SNPs *S1_87301408* and *S1_92348483* on chromosome 1 were associated with the GLS and TLB resistance, whereas *S3_205474517* SNP was associated with the SEN trait under the WS environment. Under MLN artificial inoculation, the SNPs *S3_184235364* and *S1_22259426* were among the best marker associated with MLN-DS and AUDPC values, respectively (Table 3, Table 4, Table 5 and Table 6 and Supplementary Table S2).

### 3.4. Genomic Prediction

**Figure 7:**
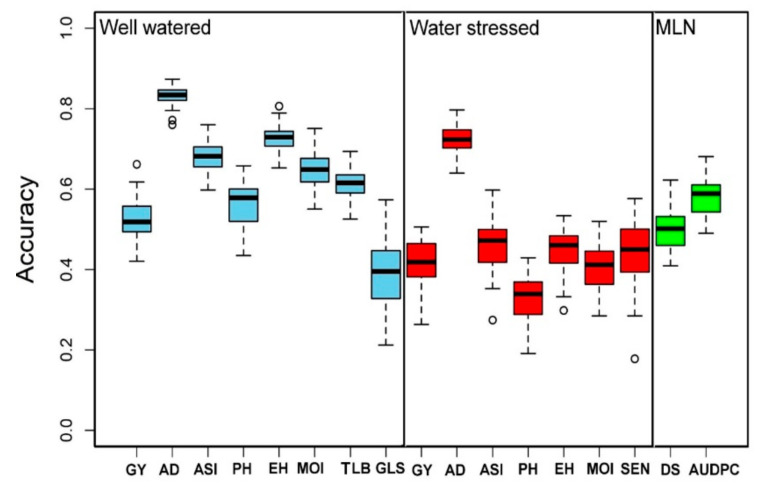
Genomic prediction accuracy results for the GY and other related traits evaluated under WW (light blue), WS (red), and MLN (green) managements. AD: anthesis date, ASI: anthesis silking interval, AUDPC: area under disease progress curve, EH: ear height, GLS: grey leaf spot, GY: grain yield, MLN-DS: maize lethal necrosis disease severity, MOI: grain moisture, TLB: turcicum leaf blight, PH: plant height, SEN: senescence, WW: well-watered, WS: water-stressed.

The selected and measured traits under WW, i.e., GY, AD, ASI, EH, PH, MOI, GLS, and TLB, had the genomic prediction accuracy values of about 0.53, 0.83, 0.68, 0.73, 0.56, 0.65, 0.39, and 0.61, respectively; while the phenotypic traits measured under WS, i.e., GY, AD, ASI, PH, EH, MOI, and SEN, had a genomic prediction accuracy values of about 0.42, 0.72, 0.46, 0.33, 0.45, 0.41, and 0.45, respectively (Figure 7). Similarly, under MLN artificial inoculation, both MLN-DS and AUDPC had 0.50 and 0.58 of genomic region prediction accuracy, respectively (Figure 7). AD depicted the highest genomic prediction accuracy under both WW and WS conditions, whereas the genomic prediction accuracy for the GY under both WW and WS environments was moderate at 0.50.

## 4. Discussion

MLN is the major challenge to maize production in SSA, specifically in east African countries. CIMMYT in collaboration with national research institutions has developed resistance breeding strategies against MLN. A large number of maize genotypes were screened, and MLN disease-resistant source materials and resistance QTLs were identified to develop resistant varieties by integrating both conventional and molecular breeding techniques [12,31,37,38]. Nevertheless, searching additional MLN disease-resistant lines, evaluation of the genotype’s performance, identification and validation of QTLs associated with the target disease, GY, and other related traits play a vital role in the development of MLN disease-resistant varieties. In this study, 879 maize DH lines derived from 26 different populations were genotyped, and the performance of genotypes were evaluated under WW, WS, and MLN artificial inoculation management conditions. Among these 879 lines, 440 DH lines shared LapostaSeqC7 background lines as one of the parent, and the line LapostaSeqC7-F64 alone used as one of the parent to develop >250 DH lines, so, data was analyzed combinedly rather making it into subgroups based analyses. 

A significant genotype, genotype by environment interaction variances, and moderate to high broad-sense heritability were observed for GY and other related traits AD, ASI, PH, EH, TLB, MOI, and GLS measured under WW and WS conditions similar with the results reported by Yuan et al. [24]. MLN-DS and AUDPC were highly heritable with 0.67 and 0.74, respectively, which is consistent with earlier reported studies [31,37,53,54]. Several genotypes have been evaluated by CIMMYT against MLN disease in search for resistant materials [12,24,31,32,38,55]; with the current study, we identified about 52 MLN disease resistant/tolerant genotypes while most of other genotypes were susceptible. Some of the maize genotypes with a score from 2 to 3 against MLN-DS were CKLMLN145667, CKLMLN145667, CKLMLN144135, CKLMLN145119, CKLMLN145173, CKLMLN143806, and CKLMLN143351, which could be selected as resistant materials to MLN disease. The mean performance of lines for GY was 7.54 t/ha and 2.7 t/ha under WW and WS environments, respectively, which has revealed a similar result in earlier study [56]. The GY had positive correlations with both EH and PH and negative correlations with ASI and MOI under WW and WS management, respectively, which could help in an indirect selection for the GY under WW and WS conditions [24].

The number of SNPs required to achieve maximum mapping resolution depends on the magnitude of LD and LD decay with genetic distance [57]. For GWAS, a large population is required since the LD or correlation between alleles in different genomic locations is generally based on the historical recombination between polymorphisms. In this study, we observed that the LD decay at r^2^ = 0.1 and 0.2 cut-offs were 10.49 and 3.69 kb, respectively. Similarly, [54] in the IMAS association panel also reported the genome-wide average LD decay of 14.97 kb at r^2^ = 0.1 and 5.23 kb at r^2^ = 0.2 [54], and a similar range of LD decay was also reported by Rashid et al. [58] in their association panel. LD decay in tropical maize germplasm was rapid compared to the temperate germplasm; possibly due to a broader genetic base, resulting from high recombination events [59]. This provides an opportunity for breeders to select germplasm that integrates high GY with disease resistance and abiotic stress tolerance.

For population structure analyses, the Delta K line plot, principal component analyses, and population genetic distance relationship analyses suggested that the utilized DH populations are structured into three to four groups. In STRUCTURE, the optimum number of subgroups was determined based on the output log-likelihood of data (LnP (D. The peaks of the line plot (Figure 4) suggest that the population could be divided into three or four distinct groups in order of possibility, with the K = 4 of delta K intersecting with LnP (D) showing a higher possibility. When K = 4, all lines were grouped as a mixed group and were further divided into three groups. The DH populations used in this study were grouped into CML395/CML505 derived DH lines, LaPostaSeq C7-F64 derived DH lines (174 individuals), and LaPostaSeq C7-F86 and LaPostaSeq F18 derived DH lines (265 individuals) (Figure 4). Due to the inclusion of DH lines derive from crosses of selected inbred lines in the panel, we observed moderate structure in the present study. Several researchers also been reported moderate structure in the tropical maize germplasm [29,31,37,53,54,60].

In this study, we identified the significant SNPs associated with target traits under WW, WS, and MLN artificial inoculations (Table 3, Table 4, Table 5 and Table 6). The results of this study for MLN-DS and AUDPC are similar to the reports in the biparental and DH population studied for the MLN-DS, AUDPC, and other traits genetic architecture [12,31,38,53,54,60]. Several putative candidate genes associated with the significant markers were identified for each of the studied traits (Table 3, Table 4, Table 5 and Table 6). For GY under WW, two putative candidate genes, *GRMZM2G017470* and *GRMZM2G030713*, were identified, both located on chromosome 1 and, respectively, described as Dof zinc finger protein DOF3.6-like and O-fucosyltransferase 36 synthesis biological functions; whereas the candidate genes, *GRMZM2G472167* on chromosome 1 and *GRMZM2G019404* on chromosome 2, identified under WS were functionally described as peptide transporter PTR2 mha2 that involved in seed germination maternal control and plasma-membrane H+ATPase 2 that aid in activating secondary transport, respectively [61,62,63]. These genes are more relevant to plants’ response to drought stress.

Putative candidate genes *GRMZM2G142383* and *GRMZM2G124136* detected for AD under WW and WS are functionally designated as Uridine kinase-like protein 2 chloroplastic involved in the pyrimidine salvage pathway [64] and putative glycerol-3-phosphate transporter 4 involved in molecular function of transmembrane transporter activity [65]. The SNPs *S8_3482389* and *S2_205904889* on chromosomes 8 and 2 were closely linked to ASI under both WW and WS associated with the putative candidate genes, *GRMZM2G136158* and *GRMZM2G105869*, respectively. These candidate genes are involved in Peroxidase 24 that aid in responding to environmental stresses such as wounding, pathogen attack, and oxidative stress [66], and histone-lysine *N*-methyltransferase SUVR3 known to be involved in the development of pollen and female gametophyte, flowering, plant morphology, and the responses to stresses [67], respectively.

The two important SNPs linked to PH *S6_161804186* under WW have shown a candidate gene *GRMZM2G170625*, and *S2_43203188* under WS, which is located with the candidate gene, *GRMZM2G114523*. Both designated candidate genes have been described as Jacalin-related lectin 3 and lysine histidine transporter-like 6 functions, respectively. Jacalin-related lectin 3 are proteins that bind carbohydrates and play an important role in plant development and resistance development to fungal pathogens [68]. Lysine histidine transporter-like 6 helps to transport amino acid within or between the cells and is involved in plant uptake of amino acids [69]. SNP, *S2_184012021* linked to EH under WW management was associated with the putative candidate gene, *GRMZM2G116196,* that was described as AUGMIN subunit 5 (AUG5) essential for the development of gametophyte and sporophyte [70] reproductions; another annotated gene *GRMZM2G365374* encoded as heat shock 70 kDa protein (HSPA1A) under WS was known to respond to heat-shock stress [71].

The SNPs *S1_188031152* and *S8_11662494* associated with the MOI were detected with well-described putative candidate genes, *GRMZM2G419436* and *GRMZM2G700386*, respectively. The gene *GRMZM2G419436* is characterized as well-associated receptor kinase 5 (WAK5), which significantly controls cell expansion, morphogenesis, and development [72], while the *GRMZM2G700386* gene characterized as β-1,2-xylosyltransferase XYXT1 is involved in the xylosylation of xylan, the primary and secondary walls or major hemicellulose of angiosperms [73]. The SNP, *S1_204865984*, linked to SEN under WS environment was the annotated putative candidate gene, *GRMZM2G328309,* explained as ribonuclease E/G-like protein, chloroplastic, which is a family of proteins that plays a pivotal function to metabolize RNA [74].

The putative genes, *GRMZM2G009591* and *GRMZM2G101117,* annotated from the SNPs *S1_246469847* and *S7_82649117* linked to GLS disease resistance had been characterized as pyrophosphate fructose 6-phosphate 1-phosphotransferase and GDSL esterase/lipase, respectively. The first gene, *GRMZM2G009591,* is known to catalyze D-fructose 6-phosphate phosphorylation [75]; the second gene, *GRMZM2G101117,* is known for the molecular function hydrolytic activities of GDSL esterases and lipases enzymes [76]. Under WW management, putative candidate genes, *GRMZM2G039173, GRMZM2G071023,* and *GRMZM2G106119* were identified based on the associated SNPs *S6_157820129* and *S4_212595942* with the TLB resistance. Rédei [77] has described the *GRMZM2G039173* gene as the major facilitator superfamily protein that aided in transporting small solutes based on the chemiosmotic ion gradients, while the second putative gene characterized by Chai et al. [78] has functioned as a probable NAD kinase 2 chloroplast, which is actively involved in the protection of chloroplast against oxidative damage and synthesis of chlorophyll.

MLN-DS trait-associated SNPs *S3_184235364* and *S6_38115747* are annotated with *GRMZM2G429982* and *GRMZM5G818106* candidate genes that have osmotin-like protein and phospholipase A1-II 7 functions, respectively [79,80]. Kumar et al. [80] characterized the candidate gene *GRMZM2G429982* as being involved in biotic and abiotic stresses tolerance in plants, whereas the candidate gene *GRMZM5G818106* has been described as protective of high temperature, cold, salt, and drought [79]. Wu et al. [81] reported that the function of the putative candidate gene *GRMZM2G003752*, which was characterized as fasciclin-like arabinogalactan protein 10, was to respond to abiotic stress and mediate the growth and development of the plant. This candidate gene was annotated from the *S2_16652265* marker associated with AUDPC values. Similarly, the *S10_125845596* marker was linked to the AUDPC value and then the putative candidate gene, *GRMZM2G003917*, was identified. This gene has been described by Wu et al. [81] as a fasciclin-like arabinogalactan protein 7 (FLA7) gene responsible for the development of microspores and, under salt stress environment, maintaining proper plant cell expansion.

In the present study, a total of 98, 54, and 22 SNPs associated with various agronomic traits under WW, WS, and MLN conditions, respectively, were identified. Among these SNPs, some existed within different gene models whose genetic role is associated with either biotic or abiotic stress mechanisms. The favorable alleles can be identified by resequencing the detected candidate genes from contrasting, and these SNPs could be potentially converted to simple PCR-based markers to follow MAS in molecular breeding [82]. Similarly, several GWAS studies reported large numbers of SNPs associated with important traits in maize [83,84].

High genetic gain can be achieved for complex traits by integrating modern tools into maize breeding [85,86]. With several genotyping service providers available with a lower cost per sample and availability of advanced statistical models, genomic prediction is routinely applied in maize for several quantitative traits [24,85,86]. In the present study, we compared the prediction accuracies under WW and WS conditions (Figure 7). As expected for all the common traits measured in both WW and WS conditions, the prediction accuracies were slightly higher under WW conditions compared to WS conditions. The observed accuracy for all traits under WW, WS, and MLN conditions reveals the effect of heritability as the traits with higher heritability generally had higher prediction accuracy. The main factors affecting genomic prediction accuracy are the relationship between the training and testing populations, training population sizes, the population structure of training and testing sets, marker densities, genetic architecture and heritability of target traits, genotype by environment interactions, and statistical methods [36,62,87,88]. Knowing the genetic architecture of the target traits, it is possible to improve prediction accuracy while implementing GS [35,89]. Moderate-to-high accuracies observed in this study for the association panel offer promise in breeding for MLN and drought tolerance. The prediction accuracy of the association panel for MLN-DS and AUDPC is in agreement with earlier studies on MLN [31] and MCMV [38]. The prediction correlations observed for GY and other agronomic traits are equivalent to earlier studies reported in maize under different stresses [24,62,85]. In GS, AD and ASI had higher accuracy compared to GY, which is expected, as these traits are less complex compared to GY [24,61,62]. GWAS results revealed GY, and other agronomic traits evaluated under WW and WS conditions are complex in nature, controlled by many loci with minor effects, influenced by environmental factors. Therefore, they are difficult to track effectively in conventional breeding alone. Increase in prediction accuracy as well as increase in accumulation of favorable alleles with both minor and major effects is possible by integration of GS with GWAS results leads.

## 5. Conclusions

Phenotypic evaluation of 879 DH lines under artificially inoculated MLN has identified about 52 genotypes resistant/tolerant to MLN-DS, while seven of the selected genotypes (CKLMLN145667, CKLMLN145667, CKLMLN144135, CKLMLN145119, CKLMLN145173, CKLMLN143806, and CKLMLN143351) can be used as sources of resistance to MLN. GWAS identified SNPs associated with the studied traits i.e., about seven and eight SNPs for the GY; 17 and 31 for anthesis date; 10 and 22 for anthesis silking interval; 14 and 6 for ear height; and 15 and 5 for moisture content under WW and WS environments, respectively. Similarly, about 13 and 11 SNPs associated with GLS and TLB, respectively, were detected. Eleven SNPs were significantly associated with senescence were identified under WS management. Under MLN artificial inoculation, a total of 12 and 10 SNPs were associated with MLN-DS and AUDPC traits, respectively; these SNPs and the identified candidate genes for each trait can be used in the trait improvement program in maize breeding. GS under WW, WS, and MLN disease artificial inoculation environments revealed moderate-to-high prediction accuracies. All the detected SNPs in this study need further validation before introducing to breeding pipelines, and it will be a great help for the understanding of complex genetic architecture traits under WW and WS. Overall, the present study identified several significant SNPs associated with GY and other agronomic traits that help in the selection of donor lines with favorable alleles for multiple traits. These results provide insights into the genetics of MLN resistance and other agronomic traits under optimum and drought stress conditions.

## Figures and Tables

**Table 1:** Estimation of mean, range, and variance components and broad-sense heritability of phenotypic traits evaluated under WW, WS, and MLN managements.

Trait	Mean (Range)	σ^2^_G_	σ^2^_GxE_	σ^2^_e_	H*^2^*	LSD_0.05_
Well-watered management						
GY	7.54 (4.35–11.66)	0.54 **	1.19 **	2.02	0.33	1.12
AD	67.72 (61.5–73.5)	2.88 **	4.14 **	2.83	0.51	3.83
ASI	0.39 (−3.0–4.5)	0.48 **	0.17 **	1.07	0.58	1.36
PH	236.21 (155.4–292.1)	73.02 **	62.05 **	191.62	0.48	18.02
EH	126.12 (79.9–169.9)	63.32 **	67.05 **	82.88	0.54	17.32
TLB	2.9 (1.0–4.9)	0.29 **	-	0.27382	0.81	1.03
MOI	23.41 (19.8–26.92)	3.00 **	0.01	7.12268	0.63	5.3
CR	1.08 (0.99–3.01)	0.02 **	-	0.05727	0.58	0.47
GLS	1.27 (0.91–2.92)	0.05 **	-	0.15261	0.57	0.77
Water stress management						
GY	2.7 (0.03–5.67)	0.21 **	-	0.64	0.40	0.78
AD	67.31 (61.1–75.5)	2.05 **	-	3.05	0.57	2.45
ASI	2.26 (−3.95–8.17)	1.61 **	-	3.02	0.52	1.84
PH	176.54 (115.5–231.8)	91.88 **	-	231.91	0.44	16.34
EH	91.26 (55.6–131.7)	28.17 **	-	82.63	0.41	12.71
MOI	12.97 (7.8–16.5)	1.02 *	-	1.55	0.57	1.1
SEN	5.96 (1.91–9.14)	0.52 **	-	1.33	0.44	0.9
ER	1.86 (0.02–51.1)	12.03 **	-	42.71	0.36	3.78
MLN management						
MLN-DS	6.02 (2.1–9.0)	1.01 **	-	0.99	0.67	0.79
AUDPC	166.47 (75.2–264.8)	642.8 **	-	453.9	0.74	20.11

H^2^: broad-sense heritability; LSD: least significant difference; *, ** significance at *p* < 0.05 and *p* < 0.01, respectively. σ^2^_G_, σ^2^_GxE_ and σ^2^_e_, represents genotypic, genotype x environment interactions and error variance, respectively. AD—anthesis date; ASI—anthesis silking interval; AUDPC—area under disease progress curve; CR—corn rust; EH—ear height; ER—ear rot; GLS—Gray leaf spot; GY—grain yield; MLN_DS—Maize lethal necrosis disease severity; MOI—moisture content; PH—plant height; SEN—senescence; and TLB—turcicum leaf blight.

**Table 2:** Number of SNPs significantly associated with grain yield and other traits at 5% false discovery rate (FDR) threshold level under well-watered (WW), water stress (WS), and MLN management.

Trait	WW	WS	MLN
GY	7	8	-
AD	16	12	-
ASI	7	8	-
EH	14	5	-
PH	14	5	-
MOI	15	5	-
GLS	14	-	-
TLB	11	-	-
SEN	-	11	
MLNDS	-	-	12
AUDPC	-	-	10
Total	98	54	22

AD—anthesis date; ASI—anthesis silking interval; AUDPC—area under disease progress curve; CR—corn rust; EH—ear height; ER—ear rot; GLS—Gray leaf spot; GY—grain yield; MLN_DS—Maize lethal necrosis disease severity; MOI—moisture content; PH—plant height; SEN—senescence; and TLB—turcicum leaf blight.

**Table 3:** Details of the GWAS results for MLN disease severity and AUDPC values associated SNP markers and their closest candidate genes identified in the large set of association mapping panel.

Trait	SNP ^a^	Chr	*p* Value	MAF	Effect	Putative Candidate Gene	Predicted Function of Candidate Gene
MLN-DS	S3_184235364	3	4.74 × 10^−8^	0.21	−0.25	GRMZM2G429982	Osmotin-like protein
	S9_139517081	9	6.93 × 10^−7^	0.36	−0.15	GRMZM2G007514	Protein SCAR2
	S7_131111643	7	9.35 × 10^−7^	0.15	−0.23	GRMZM2G467907	uncharacterized LOC100278143
	S8_160531262	8	9.51 × 10^−7^	0.31	−0.15	GRMZM2G151614	SUPPRESSOR OF ABI3-5
	S6_139620542	6	1.52 × 10^−6^	0.15	0.22	GRMZM2G061912	uncharacterized LOC100282310
	S9_3561642	9	1.79 × 10^−6^	0.18	0.19	GRMZM2G110832	uncharacterized LOC100282577
	S4_166924851	4	1.94 × 10^−6^	0.29	−0.15	GRMZM2G141036	Aspartyl protease family protein At5g10770
	S3_144742460	3	1.98 × 10^−6^	0.28	−0.13	GRMZM2G062587	Protein NETWORKED 2D
	S10_127441666	10	3.63 × 10^−6^	0.46	−0.12	GRMZM2G047370	uncharacterized LOC100280076
	S6_35895705	6	4.45 × 10^−6^	0.11	0.34	GRMZM2G035502	Glutathione dehydroascorbate reductase3
	S6_38115747	6	4.50 × 10^−6^	0.09	−0.37	GRMZM5G818106	phospholipase A1-II 7
	S3_218903424	3	6.12 × 10^−6^	0.15	0.19	GRMZM2G032244	Adenine C2 methyltransferase pseudogene
AUDPC	S1_22259426	1	3.70 × 10^−9^	0.24	4.81	GRMZM2G388915	DNA repair protein UVH3
	S9_139517081	9	7.48 × 10^−9^	0.36	−5.08	GRMZM2G007590	Spliceosomal protein
	S7_131127271	7	3.26 × 10^−7^	0.15	5.14	GRMZM2G467907	uncharacterized LOC100278143
	S10_125845596	10	3.56 × 10^−7^	0.28	−4.61	GRMZM2G003917	fasciclin-like arabinogalactan protein 7
	S4_234398586	4	3.77 × 10^-7^	0.13	−5.64	GRMZM2G111886	uncharacterized LOC100383387
	S6_139620542	6	4.23 × 10^−7^	0.15	5.86	GRMZM2G061912	uncharacterized LOC100282310
	S8_165275778	8	7.81 × 10^−7^	0.38	−3.59	GRMZM2G300375	ATP-dependent RNA helicase DHX8
	S6_38115747	6	2.25 × 10^−6^	0.09	−9.84	GRMZM5G818106	phospholipase A1-II 7
	S2_16652265	2	5.07 × 10^−6^	0.12	5.50	GRMZM2G003752	fasciclin-like arabinogalactan protein 10
	S2_3795343	2	7.82 × 10^−6^	0.27	6.20	GRMZM2G321394	protein trichome birefringence-like 8

MAF-minor allele frequency, Effect-allele effect, *p*-value probability value for the mixed linear model, MLN-DS*-*MLN disease severity, and AUDPC-area under disease progress curve values under artificial inoculation of MLN conditions; ^a^ The exact physical position of the SNP can be inferred from marker’s name, for example, S9_3561642: chromosome 9; 3,561,642 bp (Ref Gen_v2 of B73).

**Table 4:** Details of the associated SNP markers and their closest candidate genes identified in the large set of association mapping panel for GY under WW and WS conditions.

Trait	SNP ^a^	Chr	*p* Value	MAF	Effect	Putative Candidate Gene	Predicted Function of Candidate Gene
GY_WW	S1_298824055	1	2.46 × 10^−7^	0.46	−0.15	GRMZM2G017470	Dof zinc finger protein DOF3.6
	S2_213205445	2	2.17 × 10^−6^	0.24	0.16	GRMZM2G030713	O-fucosyltransferase 36
	S3_13277926	3	2.62 × 10^−7^	0.41	−0.15	GRMZM2G026783	uncharacterized
	S3_32128255	3	3.49 × 10^−7^	0.26	−0.21	GRMZM2G401294	uncharacterized
	S4_177585108	4	8.53 × 10^−7^	0.20	0.20	GRMZM2G422190	uncharacterized
	S5_206615806	5	5.79 × 10^−8^	0.34	−0.21	GRMZM2G050734	uncharacterized
	S5_208069452	5	1.03 × 10^−6^	0.39	−0.15	GRMZM2G421899	uncharacterized
GY_WS	S1_8635464	1	4.07 × 10^−6^	0.09	0.23	GRMZM2G072814	uncharacterized
	S1_285928879	1	1.07 × 10^−6^	0.09	0.23	GRMZM2G472167	peptide transporter PTR2
	S2_4269206	2	2.99 × 10^−7^	0.13	0.18	GRMZM2G019404	plasma-membrane H+ATPase 2
	S4_168744841	4	7.33 × 10^−6^	0.13	0.17	GRMZM2G422190	uncharacterized
	S4_177585059	4	5.13 × 10^−8^	0.21	0.18	GRMZM2G021339	uncharacterized
	S6_160605809	6	1.83 × 10^−6^	0.14	−0.19	GRMZM2G701221	uncharacterized
	S7_142907882	7	3.87 × 10^−6^	0.27	0.17	GRMZM2G108133	β-glucosidase 11
	S7_157468954	7	4.61 × 10^−8^	0.21	−0.20	GRMZM2G134545	dof zinc finger protein 2

MAF*-*minor allele frequency, Effect: allele effect, *p*-value: probability for the mixed linear model, GY*-*grain yield under optimum (WW) and drought stress (WS) conditions; ^a^ The exact physical position of the SNP can be inferred from marker’s name, for example, S9_3561642: chromosome 9; 3,561,642 bp (Ref Gen_v2 of B73).

**Table 5:** Details of the associated SNP markers and their closest candidate genes identified in the large set of association mapping panel for AD and ASI under WW and WS conditions.

Trait	SNP ^a^	Chr	*p* Value	MAF	Effect	Putative Candidate Gene	Predicted Function of Candidate Gene
AD_WW	S1_196052986	1	5.80 × 10^−11^	0.47	−0.3	GRMZM2G142383	Uridine kinase-like protein 2 chloroplastic
	S1_181338998	1	2.47 × 10^−8^	0.27	−0.36	GRMZM2G131254	uncharacterized LOC100191530
	S1_45513978	1	3.15 × 10^−6^	0.08	0.37	-	uncharacterized LOC100383423
	S1_77141315	1	5.83 × 10^−6^	0.14	0.37	GRMZM2G425736	uncharacterized LOC100283616
	S1_85991848	1	9.18 × 10^−6^	0.44	−0.22	GRMZM2G040743	putative calcium-dependent protein kinase family protein
	S2_164888999	2	3.56 × 10^−106^	0.06	−0.51	GRMZM2G151549	eukaryotic translation initiation factor 3 subunit 6
	S2_24033162	2	6.81 × 10^−6^	0.08	−0.39	GRMZM2G178998	uncharacterized LOC100273446
	S3_219697767	3	3.91 × 10^−6^	0.09	−0.29	GRMZM2G180815	rae1-like protein
	S4_115254061	4	1.97 × 10^−7^	0.16	0.28	GRMZM2G127690	U-box domain-containing protein 27
	S5_32847998	5	4.48 × 10^−7^	0.12	0.31	GRMZM2G130580	uncharacterized LOC100216815
	S6_135702947	6	6.59 × 10^−7^	0.13	−0.29	GRMZM2G441565	Mediator of RNA polymerase II transcription subunit 32
	S7_15457114	7	4.55 × 10^−8^	0.18	0.3	GRMZM2G028129	uncharacterized LOC100191940
	S7_172975188	7	4.84 × 10^−7^	0.12	−0.35	GRMZM2G158130	uncharacterized LOC100272539
	S7_174788925	7	1.37 × 10^−6^	0.47	−0.22	GRMZM2G006119	corticosteroid 11-β-dehydrogenase isozyme 1
	S10_88394535	10	1.09 × 10^−11^	0.3	−0.3	GRMZM5G848692	uncharacterized LOC100191174
	S10_87090061	10	8.84 × 10^−8^	0.06	0.37	GRMZM2G028104	3-*N*-debenzoyl-2-deoxytaxol N-benzoyltransferase
AD_WS	S1_70577921	1	3.41 × 10^−6^	0.28	−0.42	GRMZM2G049070	E3 ubiquitin-protein ligase EL5
	S2_28044014	2	9.88 × 10^−6^	0.46	0.31	GRMZM2G113990	coiled-coil-helix-coiled-coil-helix domain-protein 4
	S4_33885186	4	1.79 × 10^−11^	0.08	−0.69	GRMZM5G814904	catechol-*O*-methyltransferase
	S5_196250543	5	6.05 × 10^−6^	0.17	−0.33	GRMZM2G124136	Putative glycerol-3-phosphate transporter 4
	S7_127270714	7	3.75 × 10^−8^	0.31	−0.54	GRMZM2G096092	uncharacterized LOC101027214
	S7_132076042	7	7.51 × 10^−6^	0.17	0.41	GRMZM2G070375	FIP1
	S7_147051998	7	3.71 × 10^−7^	0.46	−0.32	GRMZM2G325238	putative cysteine-rich receptor-like protein kinase 35
	S7_174752364	7	2.16 × 10^−7^	0.14	0.44	GRMZM2G139870	uncharacterized LOC103633488
	S8_148392640	8	1.76 × 10^−11^	0.06	−1.01	GRMZM2G439168	E3 ubiquitin-protein ligase AIRP2
	S9_141790219	9	2.08 × 10^−6^	0.21	−0.34	GRMZM2G165357	uncharacterized LOC100193447
	S9_150809900	9	9.68 × 10^−7^	0.16	−0.45	GRMZM2G305027	D-type cyclin
	S10_85581257	10	5.47 × 10^−6^	0.49	0.3	GRMZM2G060798	uncharacterized LOC100279979
ASI_WW	S2_194040196	2	1.11 × 10^−8^	0.24	−0.2	GRMZM2G137541	uncharacterized LOC100286191
	S2_2055642	2	2.63 × 10^−6^	0.35	0.1	GRMZM2G343317	uncharacterized LOC100274748
	S2_30840909	2	9.16 × 10^−6^	0.08	0.3	GRMZM2G048366	uncharacterized LOC100194081
	S3_2218652	3	3.10 × 10^−6^	0.15	0.16	GRMZM2G026868	uncharacterized LOC100276559
	S5_53522965	5	8.54 × 10^−8^	0.22	−0.2	GRMZM2G113349	uncharacterized LOC100191684
	S6_5506422	6	7.96 × 10^−6^	0.34	−0.12	GRMZM2G450546	expansin-A19
	S8_3482389	8	2.45 × 10^−8^	0.3	0.24	GRMZM2G136158	Peroxidase 24
ASI_WS	S1_215203966	1	3.62 × 10^−6^	0.1	−0.43	GRMZM2G067235	uncharacterized LOC100275190
	S1_4748614	1	7.55 × 10^−7^	0.46	−0.26	GRMZM2G040762	uncharacterized LOC100381417
	S2_205904889	2	2.34 x 10^−6^	0.26	−0.28	GRMZM2G105869	histone-lysine *N*-methyltransferase SUVR3
	S5_211764882	5	9.75 × 10^−6^	0.14	−0.38	GRMZM2G415327	uncharacterized LOC100216930
	S9_100485294	9	4.21 × 10^−6^	0.21	−0.31	GRMZM2G104866	uncharacterized LOC100193380
	S9_136924349	9	5.99 × 10^−8^	0.2	0.38	GRMZM2G051896	nuclear poly(A) polymerase 3
	S10_135678936	10	6.72 × 10^−6^	0.44	0.3	GRMZM2G016819	Ubiquitin carboxyl-terminal hydrolase family protein
	S10_37346033	10	8.63 × 10^−6^	0.48	0.24	GRMZM2G472703	receptor kinase pseudogene

MAF-minor allele frequency, Effect*-*allele effect, *p*-value: probability for the mixed linear model, AD*-*anthesis date and ASI- anthesis silking interval under optimum (WW) and drought stress (WS) conditions; ^a^ The exact physical position of the SNP can be inferred from marker’s name, for example, S9_3561642: chromosome 9; 3,561,642 bp (Ref Gen_v2 of B73).

**Table 6:** Details of the associated SNPs and their closest candidate genes identified in the large set of association mapping panel for PH and EH under WW and WS management.

Trait	SNP ^a^	Chr	*p* Value	MAF	Effect	Putative Candidate Gene	Predicted Function of Candidate Gene
PH_WW	S1_234263371	1	2.52 × 10^−6^	0.39	1.63	GRMZM2G153233	uncharacterized LOC100304210
	S1_32087637	1	1.57 × 10^−8^	0.5	−2.04	GRMZM2G100629	uncharacterized LOC100277213
	S1_51399301	1	4.64 × 10^−8^	0.27	−2.60	GRMZM5G851485	uncharacterized LOC100274900
	S2_12352637	2	2.32 × 10^−7^	0.26	2.29	GRMZM2G156356	maltose excess protein 1-like
	S3_13264837	3	5.95 × 10^−7^	0.24	2.00	GRMZM2G026783	uncharacterized LOC100278056
	S3_166807659	3	1.52 × 10^−8^	0.39	2.30	GRMZM2G366142	uncharacterized LOC100193554
	S4_228623517	4	2.49 × 10^−6^	0.35	−1.73	GRMZM2G016923	uncharacterized LOC100502389
	S5_27226539	5	7.78 × 10^−12^	0.24	3.31	GRMZM2G428356	uncharacterized LOC100277327
	S6_161804186	6	2.08 × 10^−9^	0.25	−2.64	GRMZM2G170625	Jacalin-related lectin 3
	S7_163790932	7	2.10 × 10^−7^	0.08	−3.81	GRMZM2G106548	scarecrow-like protein 23
	S7_163967764	7	2.75 × 10^−9^	0.36	−2.32	GRMZM2G006942	exocyst complex component EXO84C
	S7_164656257	7	7.38 × 10^−6^	0.23	−1.78	GRMZM2G037545	uncharacterized LOC100217043
	S8_14844357	8	3.24 × 10^−10^	0.15	3.67	GRMZM2G052869	metallothionein-like protein 2A
	S8_67856497	8	3.49 × 10^−6^	0.09	−3.04	GRMZM2G416216	uncharacterized LOC100384078
PH_WS	S2_18001352	2	6.22 × 10^−7^	0.21	3.52	GRMZM2G318956	uncharacterized LOC100381487
	S2_43203188	2	6.87 × 10^−6^	0.15	3.09	GRMZM2G114523	lysine histidine transporter-like 6
	S7_160313368	7	3.28 × 10^−7^	0.36	−2.85	GRMZM2G431039	glucan endo-1,3-β-glucosidase 13
	S7_162003719	7	7.44 × 10^−6^	0.25	−3.11	GRMZM2G153162	eukaryotic translation initiation factor 4G
	S8_158986117	8	4.46 × 10^−9^	0.23	4.22	GRMZM2G057416	uncharacterized LOC100216812
EH_WW	S1_5744898	1	2.81 × 10^−7^	0.13	2.23	GRMZM2G025642	uncharacterized LOC100383060
	S2_184012021	2	5.92 × 10^−6^	0.22	1.4	GRMZM2G116196	AUGMIN subunit 5
	S2_54204575	2	4.86 × 10^−7^	0.22	−1.84	GRMZM2G135727	60S ribosomal protein L3
	S2_6821849	2	6.13 × 10^−7^	0.48	−1.37	GRMZM2G063519	putative galacturonosyltransferase 10
	S3_103574552	3	1.20 × 10^−6^	0.11	1.75	GRMZM2G147811	serine/threonine-protein kinase prpf4B-like
	S3_15433905	3	8.42 × 10^−11^	0.18	−2.5	GRMZM2G162182	uncharacterized LOC103651780
	S4_141091471	4	8.91 × 10^−7^	0.28	−1.56	GRMZM2G179810	Adenine phosphoribosyltransferase 2
	S4_166924899	4	3.39 × 10^−11^	0.37	2.48	GRMZM2G141036	aspartyl protease family protein At5g10770
	S4_230932334	4	2.10 × 10^−6^	0.26	−1.77	GRMZM2G004835	uncharacterized LOC100273059
	S5_182542047	5	1.39 × 10^−7^	0.49	1.69	GRMZM2G121236	umecyanin
	S5_205332507	5	1.33 × 10^−8^	0.16	2.47	-	uncharacterized LOC100502221
	S5_22544395	5	3.48 × 10^−6^	0.49	1.29	GRMZM2G059013	fringe-related protein
	S6_96126766	6	1.51 × 10^−6^	0.29	−1.41	GRMZM2G088086	uncharacterized LOC100286007
	S10_138873729	10	1.43 × 10^−7^	0.25	−1.64	GRMZM2G155776	uncharacterized LOC100276813
EH_WS	S2_195919610	2	2.45 × 10^−7^	0.44	−2.06	GRMZM2G365374	heat shock 70 kDa protein
	S2_5904286	2	2.19 × 10^−7^	0.37	−1.84	GRMZM2G372102	36.4 kDa proline-rich protein
	S7_87194068	7	2.01 × 10^−7^	0.4	−1.69	GRMZM2G157953	NAD(P)H dehydrogenase subunit CRR3 chloroplast
	S9_118825634	9	2.55 × 10^−7^	0.17	−2.24	GRMZM2G108619	uncharacterized LOC100275618
	S9_150868777	9	6.71 × 10^−6^	0.39	−1.31	GRMZM2G006721	D-type cyclin

MAF- minor allele frequency, Effect- allele effect*, p*-value probability for the mixed linear model, PH-plant height and EH-ear height under optimum (WW) and drought stress (WS) conditions; ^a^ The exact physical position of the SNP can be inferred from marker’s name, for example, S9_3561642: chromosome 9; 3,561,642 bp (Ref Gen_v2 of B73).

## Data Availability

All datasets generated for this study are included in the article. The GBS marker data are available at https://data.cimmyt.org (accessed on 2 November 2021).

## Supplementary Materials

The following supporting information can be downloaded at: https://www.mdpi.com/article/10.3390/genes13020351/s1, Figure S1: Distribution of GBS markers in the maize genome. The color key with marker densities indicates the number of markers within a window size of 1 Mb; Figure S2: Kinship heatmap generated for 879 inbred lines from 182,600 GBS SNP markers; Figure S3: Genetic relationship of 879 DH maize lines neighbor-joining tree constructed based on the population’s genetic distance matrix; Figure S4: Principal component analysis for 879 individuals with 182,600 GBS SNP markers.; Figure S5: Quantile-quantile (QQ) plot of P-values with uniform distributions. The Y-axis is the observed negative base 10 logarithms of the P-values, and the X-axis is the expected observed negative base 10 logarithms of the P-values.; Table S1: List of different DH populations and their pedigrees used in the current study; Table S2: GWAS results for Moisture content, Senescence, GLS, and TLB under well-watered and water stressed conditions.
