# Supplementary material for: Identification of Genomic Regions Associated with Agronomic and Disease Resistance Traits in a Large Set of Multiple DH Populations
Source: Genes (Basel). 2022 Feb 15;13(2):351. doi: 10.3390/genes13020351 (PMC8872035; doi:10.3390/genes13020351)
Supplement: Supplementary file 1 [file genes-13-00351-s001.zip › genes-1537456-supplementary.pdf]

genes

Supplementary Materials

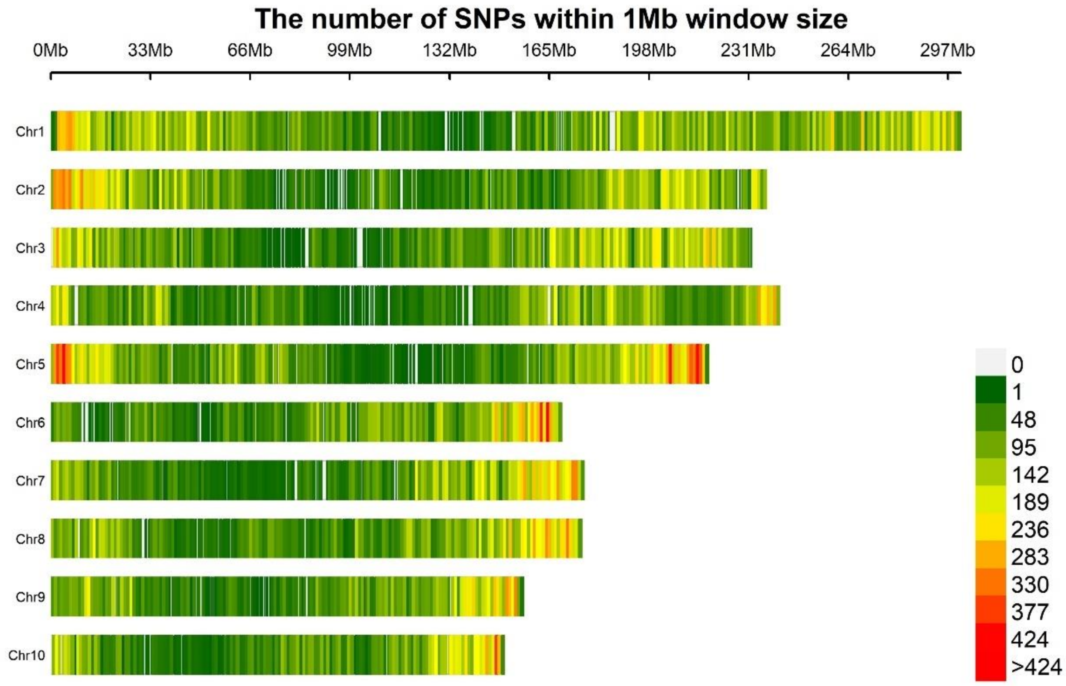

Supplementary Figure S1. Distribution of GBS markers in the maize genome. The color key with marker densities indicates the number of markers within a window size of 1 Mb.

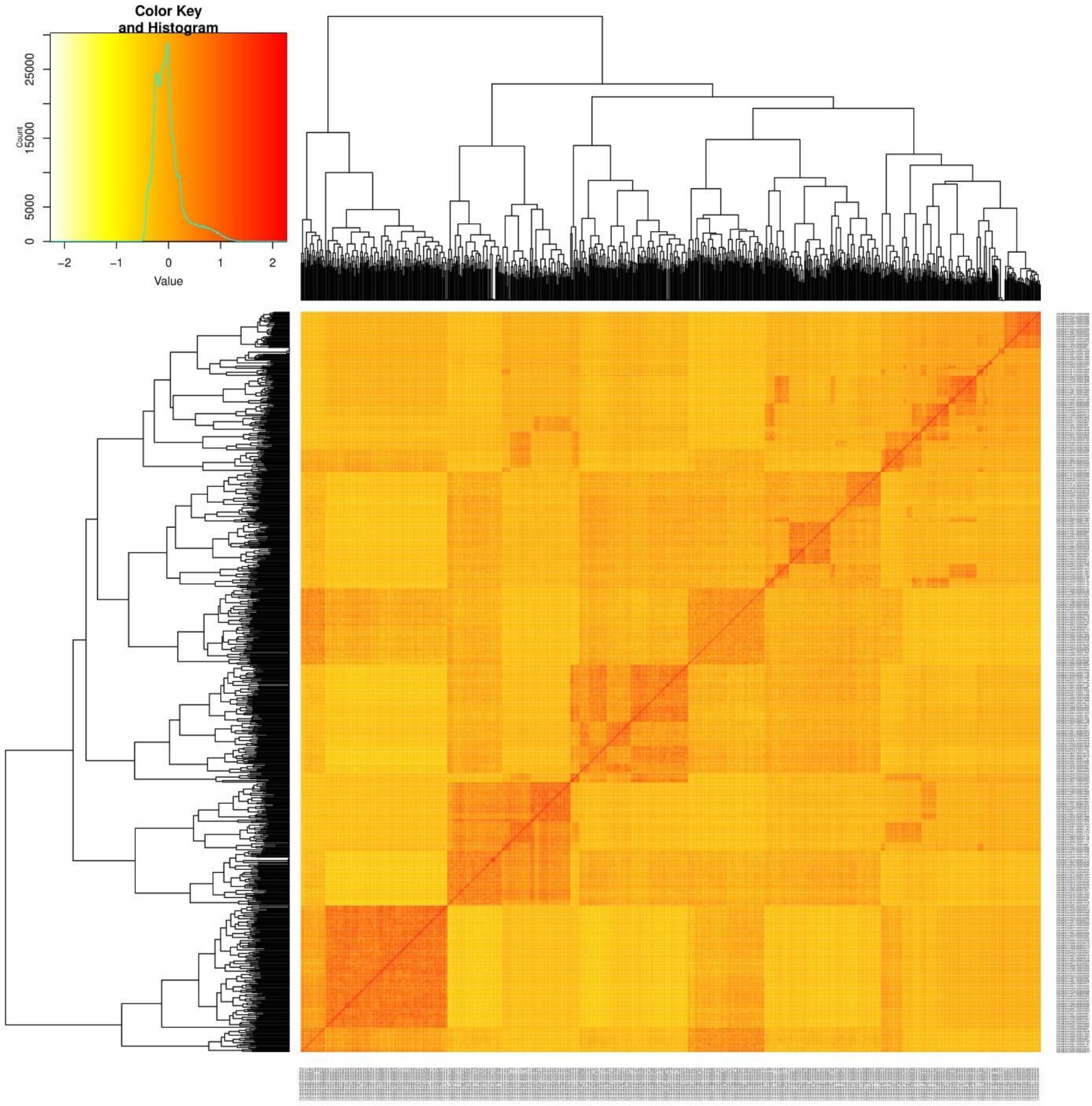

Supplementary Figure S2. Kinship heatmap generated for 879 inbred lines from 182,600 GBS SNP markers.

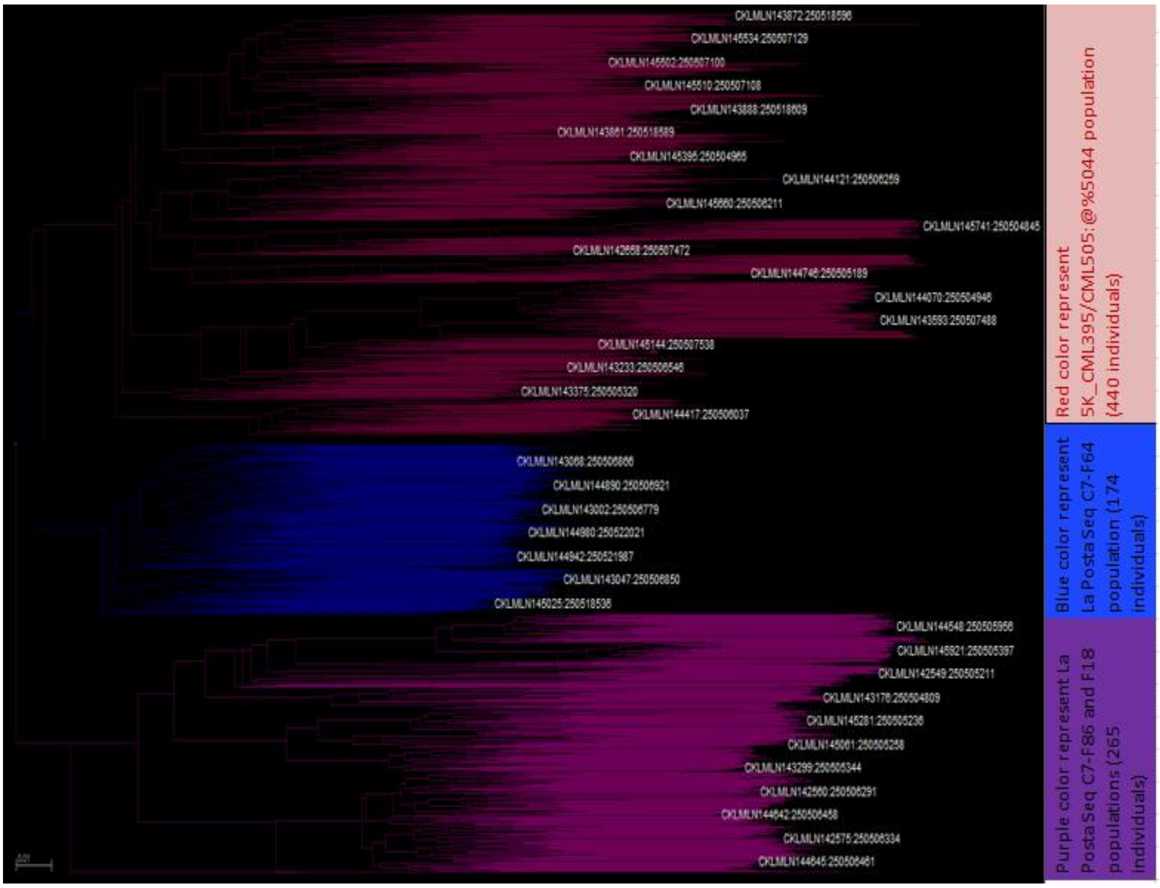

Supplementary Figure S3. Genetic relationship of 879 DH maize lines neighbor-joining tree constructed based on the population's genetic distance matrix.

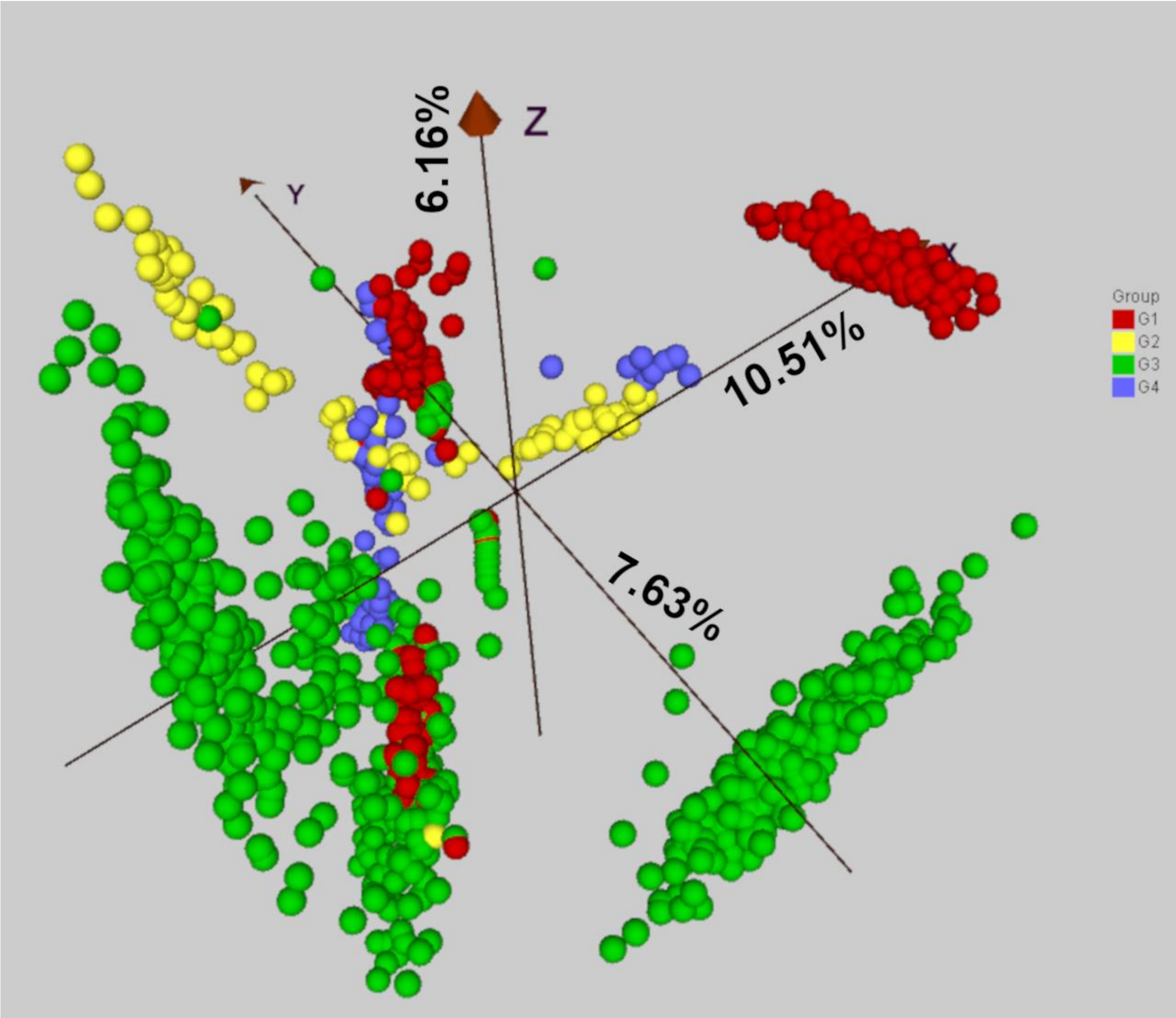

Supplementary Figure S4. Principal component analysis for 879 individuals with 182,600 GBS SNP markers. Four observed subgroups were categorized as G1, G2, G3 and G4. PC1, PC2 and PC3 explained 10.51, 7.63 and 6.16% of variations.

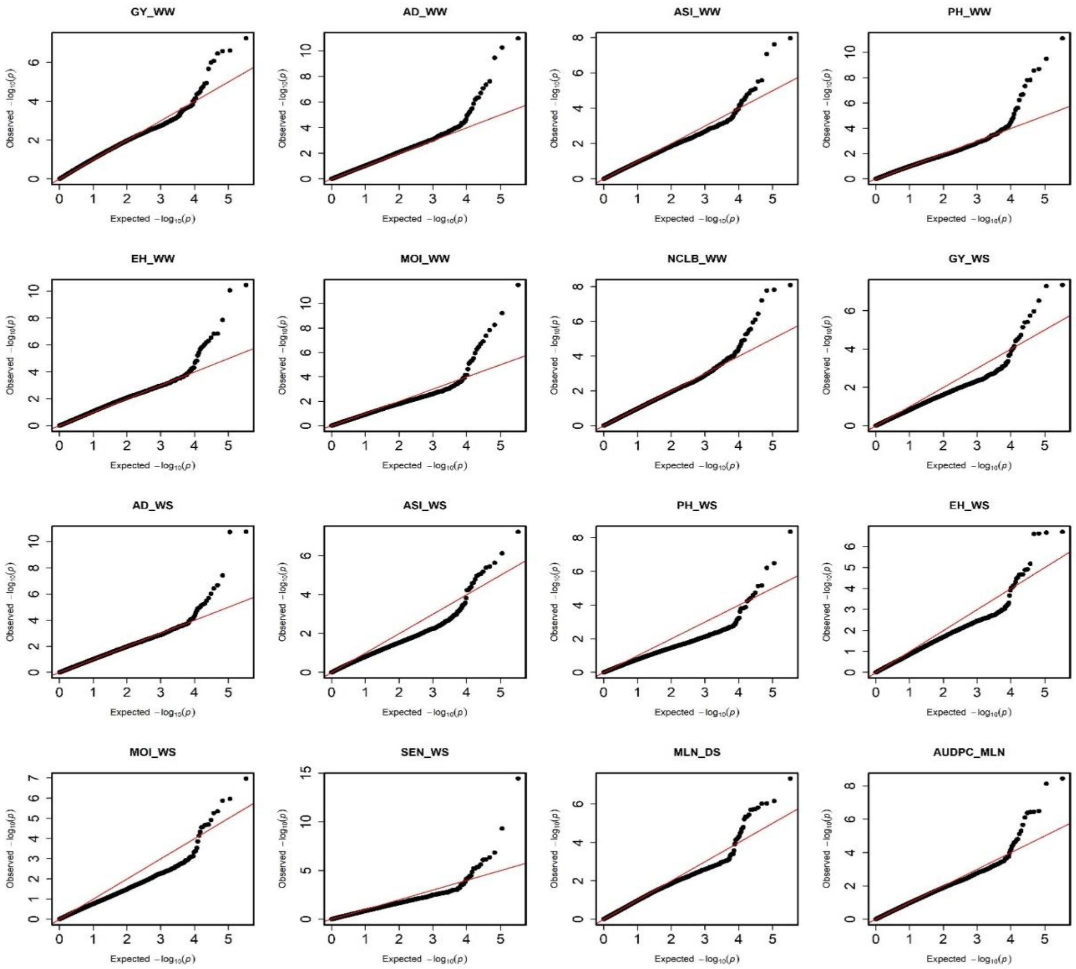

Supplementary Figure S5. Quantile-quantile (QQ) plot of P-values with uniform distributions. The Y-axis is the observed negative base 10 logarithms of the P-values, and the X-axis is the expected observed negative base 10 logarithms of the P-values. Where AD anthesis date, ASI anthesis silking interval, AUDPC area under disease pro-gress curve, EH ear height, GY grain yield, MLN-DS maize lethal necrosis disease severity, MOI grain moisture, TLB turcicum leaf blight, PH plant height, SEN senescence, WW well-watered, WS water-stressed.

Supplementary Table S1. List of different DH populations and their pedigrees used in the current study.

Pop #	Pedigree	Phenotyped	Genotyped
1	AWDH001	24	22
2	CML312/INTA-F2-192-2-1-1-1-B*7-2-B-10-B-B-B	6	-
3	CML312/LaPostaSeqC7-F18-3-2-1-1-B-B-B	12	-
4	CML312/LaPostaSeqC7-F64-2-6-2-2-B-B	54	42
5	CML312/LaPostaSeqC7-F86-3-1-1-1-B-B-B	21	-
6	CML395/CML505	182	145
7	CML440/CL-G1628=G16BNSeqC0F118-1-1-4-2-B*4-B	25	-
8	CML442/INTA-F2-192-2-1-1-1-B*7-2-B-10-B-B-B	3	2
9	CML442/LaPostaSeqC7-F18-3-2-1-1-B-B-B	25	-
10	CML442/LaPostaSeqC7-F64-2-6-2-2-B-B	53	-
11	CML442/LaPostaSeqC7-F86-3-1-1-1-B-B-B	29	14
12	CML444/CL-G1628=G16BNSeqC0F118-1-1-4-2-B*4-B	27	24
13	CML444/LaPostaSeqC7-F64-2-6-2-2-B-B	79	65
14	CML444/LaPostaSeqC7-F86-3-1-1-1-B-B-B	60	56
15	CML445/LaPostaSeqC7-F64-2-6-2-2-B-B	49	44
16	CML505/LaPostaSeqC7-F18-3-2-1-1-B-B-B	63	-
17	CML505/LaPostaSeqC7-F64-2-6-2-2-B-B	159	125
18	CML505/LaPostaSeqC7-F86-3-1-1-1-B-B-B	49	-
19	CML536/LaPostaSeqC7-F18-3-2-1-1-B-B-B	20	10
20	CML536/LaPostaSeqC7-F64-2-6-2-2-B-B	111	90
21	CML537/LaPostaSeqC7-F18-3-2-1-1-B-B-B	15	11
22	CML537/LaPostaSeqC7-F64-2-6-2-2-B-B	12	27
23	CML537/LaPostaSeqC7-F86-3-1-1-1-B-B-B	18	-
24	CML538/CL-G1628=G16BNSeqC0F118-1-1-4-2-B*4-B	15	14
25	CML538/LaPostaSeqC7-F18-3-2-1-1-B-B-B	7	7
26	CML538/LaPostaSeqC7-F64-2-6-2-2-B-B	13	13
27	CML538/LaPostaSeqC7-F86-3-1-1-1-B-B-B	2	-
28	CML539/INTA-F2-192-2-1-1-1-B*7-2-B-10-B-B-B	12	9
29	CML539/LaPostaSeqC7-F18-3-2-1-1-B-B-B	53	-
30	CML539/LaPostaSeqC7-F64-2-6-2-2-B-B	52	48
31	CML539/LaPostaSeqC7-F86-3-1-1-1-B-B-B	44	-
32	CZL04003/LaPostaSeqC7-F18-3-2-1-1-B-B-B	22	22
33	CZL04003/LaPostaSeqC7-F64-2-6-2-2-B-B	5	-
34	CZL04003/LaPostaSeqC7-F86-3-1-1-1-B-B-B	5	5
35	CZL074/VL062645	1	1
36	I-38xCML442	32	28
37	INTA-F2-192-2-1-1-1-B*7-3/LaPostaSeqC7-F18-3-2-1-1	12	5
38	RyxCML395	61	48

39	ZEWAc1F2-134-4-1-B-1-B*4-1-2-B-B/CL-G1628=G16BNSeqC0F118-1-1-4-2-B*4-B	2	2
40	ZEWAc1F2-134-4-1-B-1-B*4-1-2-B-B/LaPostaSeqC7-F64-2-6-2-2-B-B	28	-
	**Total**	**1462**	**879**

Supplementary Table S2. GWAS results for Moisture content, Senescence, GLS, and TLB under well-watered and water stressed conditions.

Trait	SNP	Chr	Position	P.value	MAF	effect	Putative Candidate gene
MOI_WW	S1_188031152	1	1.88E+08	2.99E-06	0.25	0.18	GRMZM2G419436
	S2_231382697	2	2.31E+08	1.13E-06	0.16	0.18	GRMZM2G169095
	S2_3410057	2	3.41E+06	1.44E-08	0.41	0.15	GRMZM2G047474
	S2_5904286	2	5.90E+06	8.19E-06	0.37	-0.13	GRMZM2G070881
	S3_158525333	3	1.59E+08	1.19E-07	0.15	0.23	GRMZM2G089952
	S3_219425282	3	2.19E+08	4.96E-06	0.32	-0.13	GRMZM2G143328
	S5_5111772	5	5.11E+06	5.54E-09	0.07	-0.36	Rcd1L1
	S5_5626614	5	5.63E+06	3.60E-07	0.48	0.14	GRMZM2G446213
	S8_162561752	8	1.63E+08	2.99E-12	0.06	0.47	GRMZM2G077752
	S8_171533315	8	1.72E+08	5.38E-07	0.46	0.12	GRMZM2G075023
	S8_19830105	8	1.98E+07	5.77E-10	0.09	-0.34	GRMZM2G125020
	S8_3796985	8	3.80E+06	4.19E-08	0.34	0.15	GRMZM2G044963
	S9_90842217	9	9.08E+07	4.30E-06	0.19	0.21	GRMZM2G047564
	S10_108360131	10	1.08E+08	2.00E-07	0.38	-0.18	GRMZM2G104649
	S10_134831515	10	1.35E+08	6.44E-06	0.33	0.16	GRMZM2G123450
MOI_WS	S1_33111464	1	3.31E+07	1.32E-06	0.39	-0.19	GRMZM2G147942
	S4_234405245	4	2.34E+08	4.50E-06	0.36	-0.17	GRMZM2G162992
	S5_200299111	5	2.00E+08	1.07E-07	0.15	0.33	GRMZM2G171852
	S8_11662494	8	1.17E+07	1.08E-06	0.17	0.24	GRMZM2G700386

	S9_109036121	9	1.09E+08	5.51E-06	0.29	0.18	GRMZM2G153924
SEN_WS	S1_204865984	1	2.05E+08	5.94E-06	0.47	0.11	GRMZM2G328309
	S1_279899107	1	2.80E+08	7.16E-07	0.08	-0.25	GRMZM2G159295
	S1_48689801	1	4.87E+07	4.02E-06	0.45	0.13	-
	S2_17788873	2	1.78E+07	2.35E-06	0.26	-0.13	GRMZM2G125656
	S3_172811699	3	1.73E+08	1.42E-07	0.07	-0.27	GRMZM2G105266
	S3_205474517	3	2.05E+08	3.53E-15	0.15	-0.42	GRMZM2G161868
	S4_225569867	4	2.26E+08	5.99E-06	0.11	-0.26	GRMZM2G053722; GRMZM2G423116
	S4_234916006	4	2.35E+08	4.72E-10	0.16	0.31	GRMZM2G070508
	S4_237776024	4	2.38E+08	8.03E-07	0.21	-0.17	GRMZM2G142039
	S7_141134622	7	1.41E+08	4.31E-07	0.43	0.13	GRMZM2G143955
	S7_165205564	7	1.65E+08	4.51E-06	0.22	-0.15	GRMZM2G100741
GLS_WW	S1_87301408	1	8.73E+07	1.86E-10	0.09	-0.05	GRMZM2G103668
	S1_265652951	1	2.66E+08	3.54E-07	0.09	-0.04	GRMZM6G508069
	S1_32159107	1	3.22E+07	2.49E-06	0.12	0.03	GRMZM2G100714
	S1_246469847	1	2.46E+08	9.26E-06	0.4	-0.01	GRMZM2G009591
	S2_18112313	2	1.81E+07	1.31E-09	0.1	0.05	GRMZM2G069525
	S2_126450946	2	1.26E+08	4.68E-08	0.07	0.04	GRMZM2G031802
	S3_142425041	3	1.42E+08	5.28E-06	0.12	-0.03	GRMZM2G115812
	S4_180897360	4	1.81E+08	1.36E-06	0.12	0.03	GRMZM2G300624
	S7_82649117	7	8.26E+07	4.31E-06	0.49	0.02	GRMZM2G101117
	S8_106629874	8	1.07E+08	3.69E-06	0.13	-0.02	GRMZM2G017603
	S9_87287288	9	8.73E+07	1.43E-07	0.16	0.03	GRMZM2G150323
	S9_146085650	9	1.46E+08	2.85E-06	0.09	-0.03	GRMZM2G175642
	S9_139855309	9	1.40E+08	8.62E-06	0.07	0.04	GRMZM2G700011
	S10_7156521	10	7156521	1.47255E-06	0.284982935	0.028208428	LOC109943002
TLB_WW	S1_92348483	1	9.23E+07	8.10E-09	0.1	0.16	GRMZM2G123585
	S4_212595942	4	2.13E+08	1.51E-08	0.21	-0.08	GRMZM2G071023; GRMZM2G106119
	S4_185870956	4	1.86E+08	1.13E-06	0.23	0.08	GRMZM2G439339
	S5_206151737	5	2.06E+08	5.60E-06	0.13	-0.08	GRMZM2G179147
	S6_157820129	6	1.58E+08	1.67E-08	0.38	-0.07	GRMZM2G039173
	S6_156393744	6	1.56E+08	4.84E-06	0.17	0.06	GRMZM2G094951
	S7_137487521	7	1.37E+08	2.78E-06	0.3	-0.05	GRMZM2G103713
	S8_171533315	8	1.72E+08	6.22E-08	0.46	0.05	GRMZM2G047214
	S9_55819948	9	5.58E+07	7.73E-07	0.41	0.06	New pseudogene/103638420
	S9_31283288	9	3.13E+07	3.28E-06	0.26	0.07	GRMZM2G096184
	S10_125932341	10	1.26E+08	3.68E-07	0.49	-0.07	GRMZM2G181453
